# Inclisiran-based treatment strategy in hypercholesterolaemia: the VICTORION-difference trial

**DOI:** 10.1093/eurheartj/ehaf685

**Published:** 2025-08-30

**Authors:** Ulf Landmesser, Ulrich Laufs, Ulrike Schatz, Ephraim B Winzer, Bernd Nowak, Ursula Kassner, Ioanna Gouni-Berthold, Alicia Esteban, Lawrence Lubyayi, Andre Krueger, Christian Hentschke, Andreas Wilke, Bernhard R Winkelmann, Assya Achouba, Maciej Banach

**Affiliations:** Deutsches Herzzentrum der Charité, Klinik für Kardiologie, Angiologie und Intensivmedizin, Hindenburgdamm 30, 12203 Berlin, Germany; Charité—Universitätsmedizin Berlin, corporate member of Freie Universität Berlin and Humboldt-Universität zu Berlin, Charitéplatz 1, 10117 Berlin, Germany; Department of Cardiology, Klinik und Poliklinik für Kardiologie, Universitätsklinikum Leipzig, Leipzig, Germany; Department of Internal Medicine III, Faculty of Medicine at the Technical University of Dresden, University Hospital Carl Gustav Carus, Dresden, Germany; Department for Internal Medicine and Cardiology, Heart Center Dresden, University Clinic, Technische Universität Dresden, Dresden, Germany; CCB, Cardioangiologisches Centrum Bethanien, Frankfurt a.M., Germany; Division of Lipid Disorders, Department of Endocrinology, Charité–Universitätsmedizin Berlin, corporate Member of Freie Universität Berlin and Humboldt-Universität zu Berlin, Berlin, Germany; Center for Endocrinology, Diabetes and Preventive Medicine, Faculty of Medicine and University Hospital Cologne, University of Cologne, Cologne, Germany; Clinical Development, Novartis Pharma AG, Basel, Switzerland; Advanced Quantitative Sciences, Novartis Pharmaceuticals UK Ltd, London, UK; Global Medical Affairs, Cardio Renal Metabolism, Novartis Pharma AG, Basel, Switzerland; Evidence Generation, Novartis Pharma GmbH, Nürnberg, Bayern, Germany; Kardiologische Praxis Papenburg, Papenburg, Germany; Study Center, ClinPhenomics CVC GmbH, Frankfurt, Germany; Global Medical Affairs, ASCVD, Novartis Pharma AG, Basel, Switzerland; Faculty of Medicine, the John Paul II Catholic University of Lublin, Lublin, Poland; Ciccarone Center for the Prevention of Cardiovascular Disease, Johns Hopkins University School of Medicine, Baltimore, MD, USA; Department of Preventive Cardiology and Lipidology, Medical University of Lodz, Lodz, Poland

**Keywords:** Atherosclerotic cardiovascular disease, Inclisiran, Low-density lipoprotein cholesterol, Muscle-related adverse events, Quality of life, Statins

## Abstract

**Background and Aims:**

Low-density lipoprotein cholesterol (LDL-C) is a causal risk factor for atherosclerotic cardiovascular (CV) disease development and progression. The European Society of Cardiology guidelines recommend combination treatment to achieve CV risk-based LDL-C treatment goals. Inclisiran, a small interfering ribonucleic acid (siRNA) that targets hepatic proprotein convertase subtilisin/kexin type 9 (PCSK9) messenger RNA, can provide sustained and effective LDL-C reduction.

**Methods:**

VICTORION-Difference, a phase 4 double-blind, placebo-controlled randomized clinical trial included adults with hypercholesterolaemia at high- or very high CV risk. Participants were randomized 1:1 to receive inclisiran sodium (300 mg subcutaneous injections; equivalent to 284 mg inclisiran) or placebo together with individually optimized lipid-lowering therapy (ioLLT), including up-titration with rosuvastatin (open-label) until either their individual LDL-C goal or maximally tolerated statin dose (open-label rosuvastatin) was achieved. The primary objective was assessment of LDL-C goal achievement at Day 90. Key secondary objectives were muscle-related adverse events (MRAEs) and mean LDL-C reduction. Overall, 1770 individuals (mean age, 63.7 years) were randomized to receive inclisiran (*n* = 898) or ioLLT (*n* = 872). At Day 90, a significantly higher proportion of participants receiving inclisiran vs. ioLLT achieved their individual LDL-C goals [84.9% vs 31.0%; odds ratio (OR) 12.09, *P* < .001]. The mean percentage reduction in LDL-C from baseline to Day 360 was −59.5% and −24.3% in the inclisiran and ioLLT arms, respectively [least squares mean treatment difference (LSMTD) = −35.14%, *P* < .001]. Fewer participants receiving inclisiran vs ioLLT reported a MRAE (11.9% vs 19.2%; OR 0.57, *P* < .001). The mean reduction in Short Form-Brief Pain Inventory pain severity and interference scores favoured inclisiran over ioLLT (LSMTD = −0.11, *P* = .039; LSMTD = −0.11, *P* = .029, respectively). No new safety concerns were identified.

**Results:**

Overall, 1770 individuals (mean age, 63.7 years) were randomized to receive inclisiran (n=898) or ioLLT (n=872). At Day 90, a significantly higher proportion of participants receiving inclisiran vs. ioLLT achieved their individual LDL-C goals (84.9% vs. 31.0%; odds ratio [OR] 12.09, p<0.001). The mean percentage reduction in LDL-C from baseline to Day 360 was -59.5% and -24.3% in the inclisiran and ioLLT arms, respectively (least squares mean treatment difference [LSMTD]=-35.14%, p<0.001). Fewer participants receiving inclisiran vs. ioLLT reported a MRAE (11.9% vs. 19.2%; OR 0.57, p<0.001). The mean reduction in Short Form-Brief Pain Inventory pain severity and interference scores favoured inclisiran over ioLLT (LSMTD=-0.11, p=0.039; LSMTD=-0.11, p=0.029, respectively). No new safety concerns were identified.

**Conclusions:**

An inclisiran-based treatment strategy was superior to ioLLT in LDL-C goal achievement, delivering early and sustained LDL-C reduction, with fewer MRAEs in individuals with hypercholesterolaemia.

## Structured Graphical Abstract

**Structured Graphical Abstract:**
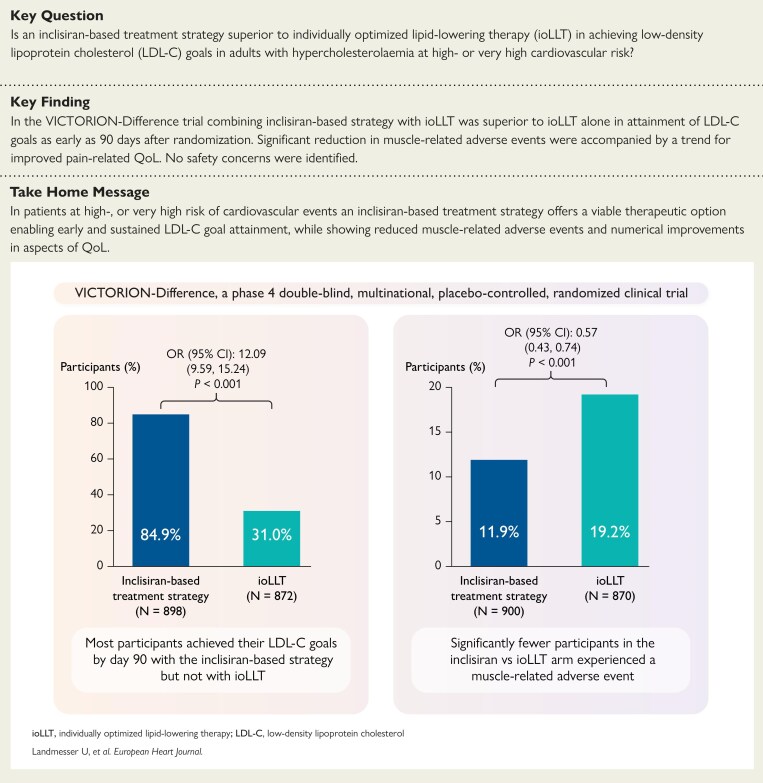

**See the editorial comment for this article ‘In pursuit of LDL-C goal attainment: the VICTORION-Difference trial and the dawn of proactive lipid management’, by K. Miteva and F. Mach, https://doi.org/10.1093/eurheartj/ehaf1009.**

## Introduction

Cardiovascular disease (CVD) is a major global healthcare concern^1^ and accounted for 20.5 million deaths worldwide in 2021, representing a 1.7-fold increase compared with 1990.^2^ This growing prevalence places a substantial burden on affected individuals and healthcare systems, highlighting the urgent need for effective prevention and management strategies.^3,4^ Patients with CVD consistently exhibit impaired health-related quality of life, encompassing physical, social and economic dimensions,^3^ and have a substantially increased risk of future cardiovascular (CV) events.^5^

Long-term exposure to persistently elevated levels of low-density lipoprotein cholesterol (LDL-C) is causal in atherosclerotic CVD (ASCVD),^6–8^ with every 1 mmol/L (∼39 mg/dL) reduction lowering the risk of CV events by 20%–25%.^9^ Consequently, multiple global clinical guidelines and expert consensus statements recommended strategies to reduce the cumulative exposure to LDL-C, which includes the routine use of combination lipid-lowering therapies (LLTs), and statins as essential medications.^8,10–12^ Despite available medications and treatment guidelines recommending statin therapy as the first-line treatment for LDL-C reduction,^8^ many patients on statins fail to achieve the recommended LDL-C goals.^13–15^ Indeed, findings of a cross-sectional observational study conducted in 18 countries across Eastern Europe, Asia, Africa, the Middle East and Latin America demonstrated that only 32% of very high-risk patients achieved their LDL-C goals.^14^ Concerns about side effects, especially statin-associated muscle symptoms (SAMS), lead many patients to view statins as intolerable, reducing adherence,^16–19^ which in turn elevates CV risk.^20^ Real-world evidence indicates that women are more likely than men to exhibit poor adherence to statin therapy, with a higher tendency to discontinue treatment due to adverse effects.^21,22^ Although statins are a cornerstone in the management of ASCVD, their use remains suboptimal, at least in part, due to concerns of muscle-related adverse effects (MRAEs).^23^ While MRAEs are most commonly associated with statin use, they are not exclusive to this class of drugs.^24^ Moreover, the incidence of reported adverse effects with statins may, in part, be influenced by the nocebo effect, wherein negative expectations contribute to the perception or experience of side effects.^23,25^ Nonetheless, SAMS may have a considerable impact on patients quality of life (QoL), with a substantial proportion reporting major disruption of their everyday life.^26,27^ This highlights the need for effective treatment options that can achieve robust reductions in LDL-C levels while minimizing MRAEs to improve both clinical outcomes and QoL.

Inclisiran, a small interfering ribonucleic acid (siRNA) that targets hepatic proprotein convertase subtilisin/kexin type 9 (PCSK9) messenger RNA, provides consistent reductions in LDL-C levels with twice-yearly administration, following the initial and 3-month doses.^28^ Results of a long-term study demonstrated that with a cumulative treatment duration of up to 6.8 years, twice-yearly administration of inclisiran provided sustained and effective LDL-C reduction of 49.4% in patients with high CV risk, with a good long-term safety profile.^29^ Despite substantial evidence from clinical trials demonstrating the safety and efficacy of inclisiran,^30^ its impact on the QoL of patients with ASCVD when utilized as an LDL-C-lowering treatment strategy on top of ongoing LLT remains unknown. VICTORION-Difference is the first study designed to better understand participants’ perspectives on satisfaction with ongoing individually optimized LLT (ioLLT) with or without inclisiran and impairment due to statin-related muscle pain using patient-centric QoL measures as secondary endpoints. The study investigated the efficacy of inclisiran compared with placebo, both administered on top of ongoing CV risk-based ioLLT according to treatment guidelines, in achieving guideline-recommended LDL-C goals, and impact on MRAEs, pain-related QoL scores and mean LDL-C reduction.^31^

## Methods

### Trial design

The design and methodology of the VICTORION-Difference study (NCT05192941) have been published previously.^31^ In brief, this was a phase 4 randomized, double-blind, placebo-controlled clinical trial, conducted in 133 sites across eight European countries (Bulgaria, Czech Republic, Estonia, France, Germany, Latvia, Poland, and Spain).

Participants were screened based on LDL-C levels while receiving a maximally tolerated dose (MTD) of statins. The study aimed to randomize individuals who remained uncontrolled despite optimized statin therapy; therefore, participants who achieved LDL-C targets while on their MTD were excluded. The study was designed to ensure that each participant was on a stable statin dose, maintained for ≥30 days prior to the screening visit. This dose was further optimized during the run-in period and the double-blind phase. The statin dose was sequentially and optimally titrated to the MTD until the participants achieved either their individual LDL-C goals or their MTD of statin. Those already on an MTD for ≥30 days and still uncontrolled were randomized directly. Participants not yet at MTD entered a run-in phase to optimize statin dosing and reassess LDL-C levels. This phase, which could last up to 120 days, allowed sufficient time for dose titration and evaluation of eligibility for randomization. At baseline, all enrolled participants were randomized 1:1 to receive double-blind subcutaneous injections of inclisiran sodium (300 mg; equivalent to 284 mg inclisiran) or placebo on top of ioLLT, with subsequent doses administered at days 90, and 270. In addition to receiving inclisiran or placebo, participants were administered open-label rosuvastatin at randomization and during subsequent visits according to the local summary of product characteristic (SmPC). The starting dose of open-label rosuvastatin was 5 mg/day or 10 mg/day. Based on the local SmPC, the investigator selected an appropriate starting dose of rosuvastatin for each participant. If the investigator determined that neither of the recommended starting doses of rosuvastatin (5 mg or 10 mg once daily) was appropriate for a participant, the individual was not enrolled in the study. The distribution of starting doses used at baseline is detailed in the supplementary materials. Participants who were already receiving rosuvastatin prior to baseline continued their individual MTD, as deemed appropriate by the investigator. At randomization, all participants were receiving their statin MTD and were switched to rosuvastatin at a standard starting dose. Following randomization and the switch to rosuvastatin, achievement of MTD varied between groups but was reached in the majority of participants. As this initial dose was not equivalent to the participant’s prior MTD, rosuvastatin was titrated every 4 weeks as needed. If LDL-C targets were not achieved despite reaching the MTD of rosuvastatin, additional LLTs were introduced sequentially, following the study’s escalation protocol. This stepwise approach continued throughout the follow-up period, reflecting ongoing LLT optimization over the course of 1 year. The participant's MTD of statin was defined as either the highest approved dose of a respective statin (as per local SmPC) or the other maximum dose of statin that can be taken by the participant on a regular basis without intolerable AEs that required dose adjustment, or without AEs that required a dose adjustment (e.g. elevated lab values as per local SmPC), or without meeting any contraindication for a particular participant (as per local SmPC).

The primary objective of the study was to assess whether an inclisiran-based treatment strategy (inclisiran + ioLLT) was superior to placebo administered on top of ongoing CV risk-based ioLLT in achieving LDL-C goals, as defined by the 2019 ESC/EAS guidelines^8^ for the management of dyslipidaemias, by Day 90. The key secondary objectives were evaluating the superiority of an inclisiran-based treatment strategy vs ioLLT on MRAEs and the mean LDL-C reduction over the double-blind study period, with an annualized number of days with pain and pain-related QoL as other secondary objectives. Details related to the trial design, population and assessments were published previously^31^ and briefly summarized in the Supplementary data online, methods.

### Access to LLTs and escalation strategy

The study design included a feasibility phase, during which a survey was conducted across participating countries to evaluate the availability and reimbursement status of LLTs, excluding rosuvastatin, which was already provided by the sponsor as an auxiliary medication. The survey confirmed that ezetimibe and cholestyramine—both generic drugs—were widely accessible, and no significant access issues were reported, with the only anticipated challenge being potential tolerability issues with cholestyramine. Bempedoic acid, designated as the escalation step following statins, ezetimibe, and cholestyramine, received approval from the European Medicines Agency in April 2020, while the study continued from 8th April 2022 (first patient first visit) to 19th March 2025 (last patient last visit). No major access issues were anticipated across participating countries, particularly in Germany, which was the primary contributor to patient enrolment. In instances where a LLT could not be added during the escalation phase—due to availability or reimbursement constraints—the medication was sourced locally, and the cost was covered by the study sponsor. Participants who failed to achieve LDL-C goals despite receiving the full treatment regimen were potentially eligible for PCSK9 monoclonal antibodies (mAbs), subject to a rigorous approval process. The principal investigator submitted a PCSK9 mAb request form to the sponsor for review to ensure all required information was complete. A dedicated board of independent experts reviewed each PCSK9 mAb request using a standardized form (see Supplementary data online, *Table S1*). The board assessed the participant’s CV risk profile and treatment history, ensuring that all protocol-defined LLT steps—including rosuvastatin titration to MTD, and sequential addition of other LLTs—had been completed. If any step was omitted, a documented rationale was required. Unlike other LLTs, access to PCSK9 mAbs is highly restricted across Europe. Upon approval by the expert board, PCSK9 mAbs were provided by the sponsor, and the study drug was discontinued. This adaptive strategy was implemented to proactively address potential LLT access issues, ensuring equitable treatment escalation across all study sites, even in the absence of reported restrictions during the feasibility phase.

### Trial population

The trial included participants aged ≥18 years with hypercholesterolemia at high or very high CV risk, according to the 2019 ESC/EAS guideline^8^ and updated SCORE2 and SCORE2-older persons.^32,33^ Participants were required to be on a stable dose of a statin for ≥30 days and have a fasting triglyceride of <400 mg/dL (<4.6 mmol/L). Key exclusion criteria were described previously.^31^ For those receiving ≥1 LLT on top of statins, enrolment was capped so that ∼20% of randomized participants received a stable dose of an additional LLT alongside statin therapy at the screening visit, reflecting current and anticipated prescribing practices. Participants were categorized as high- or very high-risk individuals at baseline based on medical history and other assessments, including LDL-C levels [very-high risk: ≥55 mg/dL (>1.4 mmol/L)]; [high-risk: ≥70 mg/dL (>1.8 mmol/L)].

Supplementary data online, *Table S2* provides the full list and description of patient-reported outcomes assessed during the VICTORION-Difference study, including SF-BPI and pain diary for which data are presented in this manuscript.

### Endpoints

The primary endpoint was the proportion of participants achieving guideline-recommended individual LDL-C goals [very-high CV risk: <55 mg/dL (<1.4 mmol/L); high CV risk: <70 mg/dL (<1.8 mmol/L)] evaluated at Day 90. Key secondary endpoints included: (i) relative change (percentage change) from baseline in LDL-C level over the double-blind study period (averaged over all post-baseline visits); and (ii) the proportion of participants experiencing at least one MRAE from baseline Day 1 to Day 360 [as defined in the Standardized MedDRA Query (SMQ) rhabdomyolysis/myopathy]. Other secondary endpoints included (i) the annualized mean number of days participants experiencing self-reported pain from baseline to Day 360 (assessed using a pain diary); (ii) change from baseline in Short-Form Brief Pain Inventory (SF-BPI) pain severity and interference scores at Day 360; and (iii) the proportion of participants with clinically relevant change in SF-BPI pain severity and interference score from baseline to Day 360. Other efficacy assessments included assessment of total cholesterol, high-density lipoprotein cholesterol (HDL-C), non-HDL-C, triglycerides, apolipoprotein B (Apo B), lipoprotein(a) (Lp[a]), and PCSK9 levels.

Safety assessments included monitoring of AEs [including treatment-emergent AEs (TEAEs) and AEs of interest], serious AEs, and laboratory markers in blood and urine.

Prior to conducting any clinical assessments, participants also completed the patient-reported outcome measures(s) at scheduled visits, which included the SF-BPI and pain diary to assess pain-related QoL.

### Statistical analysis

Sample size calculations ensured that the study was powered for the primary endpoint, proportion of participants achieving guideline-recommended individual LDL-C goals at Day 90, and for the secondary endpoint: proportion of participants experiencing ≥1 MRAE from Day 1 to Day 360. For the primary and secondary efficacy endpoints, missing values were handled using multiple imputation (see supplementary materials for more details). The intercurrent event of discontinuation of study treatment was handled with the treatment policy strategy i.e. follow-up information after permanent discontinuation of study treatment, retrieved drop out data collected at regular study visits were included in the analysis, with treatments as assigned at randomization. For death, a while alive strategy was considered i.e. the last value measured before death was included in the analysis. For use of PCSK9 targeting non-study medication, or lipid apheresis, the composite strategy was employed: participants who used PCSK9 targeting non-study medication or lipid apheresis were considered as not achieving their LDL-C target.

A logistic regression model, adjusted for CV risk category, was used to assess the odds ratio [OR; 95% confidence interval (CI)] for achieving individual LDL-C targets for the primary analysis and for MRAEs. A one-sided *P*-value of .025 was considered significant. Mixed models for repeated measures (MMRMs) were utilized to analyse the relative change from baseline in LDL-C levels and the absolute change from baseline in SF-BPI pain severity and pain interference scores over the double-blind treatment period. The proportion of participants experiencing at least one muscle-related AE from Day 1 to Day 360 was analysed using a logistic regression model, while a negative binomial model was utilized to assess the annualized number of pain days experienced. All efficacy variables were analysed using the full analysis set, which included all randomized participants who received ≥1 dose of double-blind study medication. Safety summaries were presented by treatment groups for all data from the safety set, which included all participants who received at least one dose of double-blind study treatment.

A hierarchical testing strategy was employed to analyse the primary and secondary endpoints to strictly control the rate of familywise type I error. To fit into the confirmatory testing framework of primary and secondary hypotheses, a one-sided *P*-value of <.025 was considered significant. The six hypotheses were grouped into three families. The first family contained the primary hypothesis, H01. The second family contained two secondary hypotheses: percentage change from baseline in LDL-C over the double-blind study period (H02) and the proportion of participants experiencing at least one MRAE (H03). The third family contained the rest of the secondary hypotheses: annualized mean number of days participants experienced self-reported pain from baseline to Day 360 (H04), absolute change in SF-BPI pain severity (H05) and interference (H06) scores from baseline to Day 360. The hierarchical testing strategy was applied to these three families. First H01 was tested at level alpha. If H01 was rejected, then the second family followed with a closed test at level alpha. If both H02 and H03 were rejected, then the third family was tested using the Hommel procedure at level alpha. When not all hypotheses in a family could be rejected, testing would stop at the corresponding family and would not proceed to the next family. Statistical Analysis Software version 9.4 was used for the analyses.

## Results

### Baseline characteristics

Of the 2529 individuals screened, 1770 were randomized to receive study medication (inclisiran, *n* = 898; ioLLT, *n* = 872); thus 30% of the participants discontinued prior to randomization (see Supplementary data online, *Figure S1*). For additional data on participant disposition, see Supplementary data online, *Tables S3* and *S4*. Overall, 97.1% of participants completed the study, with subject decision being the most common reason for discontinuation across both arms (inclisiran, *n* = 8 of 18; ioLLT, *n* = 15 of 18). The overall demographic and clinical characteristics of the study participants at baseline were comparable across the two treatment arms (*Table 1* and Supplementary data online, *Table S5*). The mean [standard deviation (SD)] age of participants was 63.7 (9.84) years, with 48.5% aged 45–<65 years. The majority of the participants were men (69.8%), and 96.9% were white. Eight countries (all European) were involved in the study, and most participants were recruited from Germany (55.5%), followed by Bulgaria (11.4%) and Czech Republic (6.8%) (see Supplementary data online, *Table S6*). The baseline median LDL-C level of the study population was 2.18 mmol/L.

**Table 1:** Demographics and baseline characteristics of participants included in the VICTORION-difference study (full analysis set)

Characteristic Statistic/category	Inclisiran-based treatment strategy *N* = 898	ioLLT *N* = 872	Total *N* = 1770
Age (years)			
*N*	898	872	1770
Median	64	64	64
Q1–Q3	57–70	58–70	58–70
Race—White, *n* (%)	870 (96.9)	845 (96.9)	1715 (96.9)
Sex, *n* (%)			
Male	625 (69.6)	610 (70.0)	1235 (69.8)
Female	273 (30.4)	262 (30.0)	535 (30.2)
BMI (kg/m^2^)			
*N*	898	872	1770
Median	29.4	29.0	29.1
Q1–Q3	26.1–33.1	26.22–32.7	26.2–32.9
Alcohol history, *n* (%)			
Current	529 (58.9)	485 (55.6)	1014 (57.3)
Never	316 (35.2)	321 (36.8)	637 (36.0)
Former	53 (5.9)	66 (7.6)	119 (6.7)
Smoking history, tobacco, *n* (%)			
Current	203 (22.6)	216 (24.8)	419 (23.7)
Never	314 (35.0)	306 (35.1)	620 (35.0)
Former	381 (42.4)	350 (40.1)	731 (41.3)
LDL-C level (mmol/L)			
*N*	898	872	1770
Median	2.18	2.12	2.18
Q1–Q3	1.81–2.69	1.78–2.67	1.79–2.69
ApoB (g/L)			
*N*	898	872	1770
Median	0.85	0.85	0.85
Q1–Q3	0.74–1.02	0.74–1.00	0.74–1.01
HDL-C (mmol/L)			
*N*	898	872	1770
Median	1.30	1.29	1.30
Q1–Q3	1.06–1.58	1.06–1.55	1.06–1.55
Non-HDL-C (mmol/L)			
*N*	898	872	1770
Median	2.87	2.82	2.85
Q1–Q3	2.41–3.50	2.38–3.47	2.41–3.47
Total cholesterol (mmol/L)			
*N*	898	872	1770
Median	4.27	4.20	4.25
Q1–Q3	3.78–4.92	3.70–4.84	3.76–4.87
Trigycerides (mmol/L)			
*N*	898	872	1770
Median	1.36	1.36	1.36
Q1–Q3	0.97–1.93	1.03–1.86	1.01–1.90
CV risk category, *n* (%)			
Very high risk	822 (91.5)	811 (93.0)	1633 (92.3)
High risk	76 (8.5)	61 (7.0)	137 (7.7)
Comorbidities			
Diabetes mellitus	364 (40.5)	359 (41.2)	723 (40.8)
Heart failure	191 (21.3)	200 (22.9)	391 (22.1)
Hypertension	756 (84.2)	751 (86.1)	1507 (85.1)
Ischemic stroke	72 (8.0)	70 (8.0)	142 (8.0)
Myocardial infarction	344 (38.3)	372 (42.7)	716 (40.5)
Percutaneous coronary intervention	424 (47.2)	450 (51.6)	874 (49.4)
Peripheral artery disease	145 (16.1)	127 (14.6)	272(15.4)
Stable angina	205 (22.8)	208 (23.9)	413 (23.3)
Unstable angina	98 (10.9)	104 (11.9)	202 (11.4)
Systolic blood pressure (mmHg)			
*N*	898	872	1770
Median	132	132	1327
Q1–Q3	122–141	122–142	122–141
Diastolic blood pressure (mmHg)			
*N*	898	872	1770
Median	80	80	80
Q1–Q3	74–85	73–85	73–85
Creatine kinase (U/L)			
*N*	898	872	1770
Median	108	108	108
Q1–Q3	75–161	77–162	76–161

ApoB, apolipoprotein B; BMI, body mass index; CV, cardiovascular; eGFR, estimated glomerular filtration rate; HbA1c, hemoglobin A1c; HDL-C, high-density lipoprotein cholesterol; ioLLT, individually optimized lipid-lowering therapy; LDL-C, low-density lipoprotein; Q, quartile.

Overall, 7.7% and 92.3% of participants were classified as having high and very high CV risk, respectively (*Table 1*). The mean (SD) exposure to an inclisiran-based treatment strategy and ioLLT during the double-blind treatment period was 360.4 (36.07) and 357.6 (42.6) days, respectively. The cumulative exposure (SD) to rosuvastatin was 347.9 (61.8) and 346.0 (65.7) days for participants receiving an inclisiran-based treatment strategy and ioLLT, respectively.

### Efficacy

#### Primary endpoint

**Figure 1:**
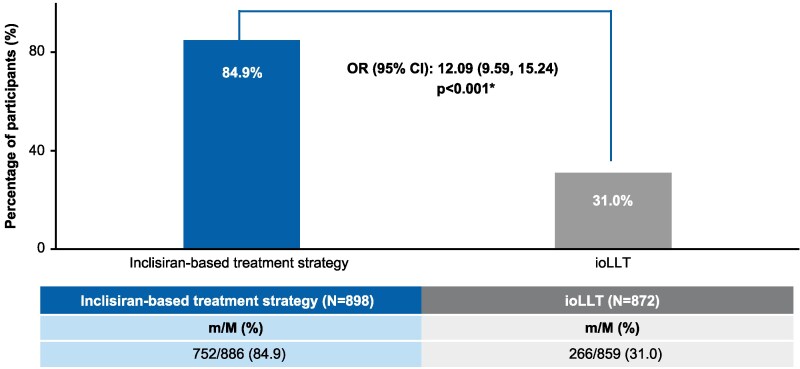
Proportion of participants achieving their individual LDL-C goals at day 90 in the VICTORION-difference study (full analysis set). *Significant according to testing hierarchy (1-sided *P*-value). CI, confidence interval; LDL-C, low-density lipoprotein cholesterol; ioLLT, individually optimized LLT; LLT, lipid-lowering therapy; m, number of participants who achieved their individual LDL-C target [<55 mg/dL (<1.4 mmol/L) for very high CV risk group or <70 mg/dL (<1.8 mmol/L) for high CV risk group] at Day 90; M, number of participants with an LDL-C value at Day 90; OR, odds ratio

**Figure 2:**
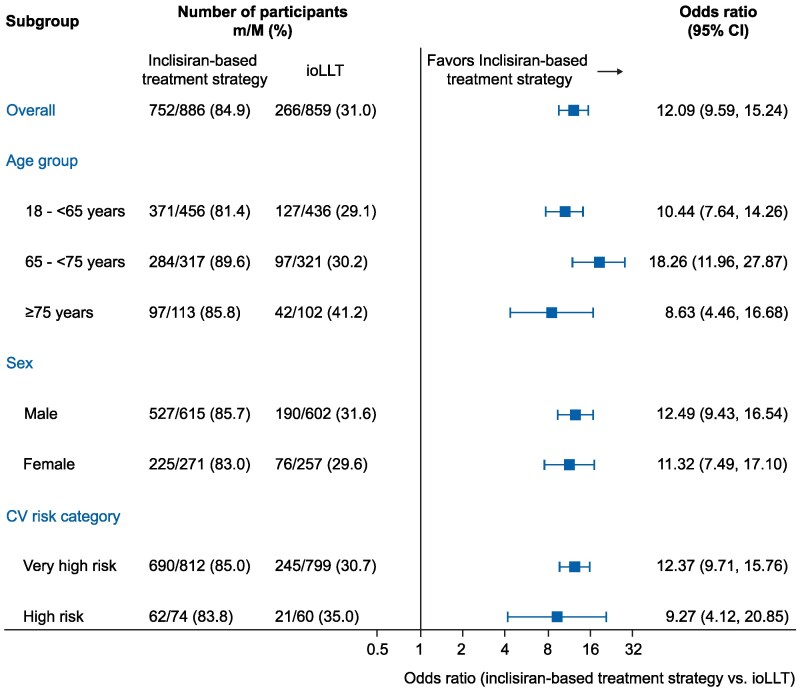
The odds ratio for achieving individual LDL-C goals at day 90 in the VICTORION-difference study by subgroup (full analysis set). CI, confidence interval; CV, cardiovascular; LDL-C, low-density lipoprotein cholesterol; ioLLT, individually optimized LLT; LLT, lipid-lowering therapy; M, the number of participants who achieved their individual LDL-C target (< 55 mg/dL or <70 mg/dL) at Day 90; M, the number of participants with an LDL-C value at Day 90; m/M are considering intercurrent event strategy but not multiple imputation

A significantly higher proportion of participants treated with inclisiran vs ioLLT achieved their individual LDL-C goals at Day 90 (84.9% vs 31.0%; OR 12.09; 95% CI: 9.59, 15.24; *P* < .001; *Figure 1*). Across all participant subgroups, the odds of achieving LDL-C goals by Day 90 were significantly higher among those treated with an inclisiran-based treatment strategy vs ioLLT (*Figure 2*).

#### Secondary endpoints

##### LDL-C reduction

**Figure 3:**
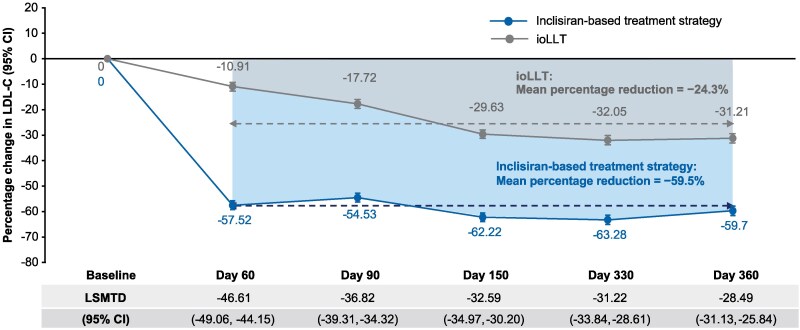
Percentage change in mean LDL-C levels (95% CI) from baseline over the double-blind study period in the VICTORION-difference study (full analysis set). Mean percentage change from baseline over the double-blind study period (averaged over all post-baseline visits). CI, confidence interval; LDL-C, low-density lipoprotein cholesterol; ioLLT, individually optimized LLT; LLT, lipid-lowering therapy; LSMTD, least squares mean treatment difference

Despite participants in both treatment arms undergoing individualized titration of rosuvastatin and addition of other LLTs, the mean percentage reduction of LDL-C from baseline to Day 360 was significantly greater with an inclisiran-based treatment strategy vs ioLLT (−59.5% vs −24.3%; *Figure 3*), resulting in a between-group difference of (35.14%; 95% CI: −37.07, −33.22; *P* < .001). This greater reduction was seen from Day 60 (−57.5% vs −10.9%) and sustained throughout the trial period until Day 360 (−59.7% vs 31.2%).

Additional analyses revealed that more participants treated with an inclisiran-based strategy consistently achieved their individual LDL-C goals vs ioLLT at each visit, which was evident as early as Day 30 (80.8% vs 11.3%; Supplementary data online, *Figure S2*).

##### MRAEs and pain-related QoL

**Figure 4:**
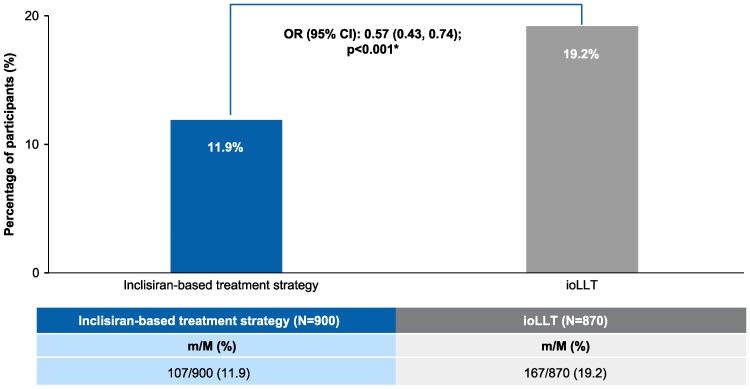
Proportion of participants experiencing at least one muscle-related AE from day 1 to day 360 in the VICTORION-difference study (safety set). *Significant according to testing hierarchy (1-sided *P*-value). CI, confidence interval; LDL-C, low-density lipoprotein cholesterol; ioLLT, individually optimized LLT; LLT, lipid-lowering therapy; m, number of participants who experienced at least one muscle-related adverse event, defined as SMQ rhabdomyolysis/myopathy from Day 1 to Day 360 (all MRAEs occurring between first dose of double-blind treatment and earliest date out of visit Day 360, death date and last contact date are considered); M, number of participants in the safety set; OR, odds ratio; SMQ, standardized MedDRA queries

**Figure 5:**
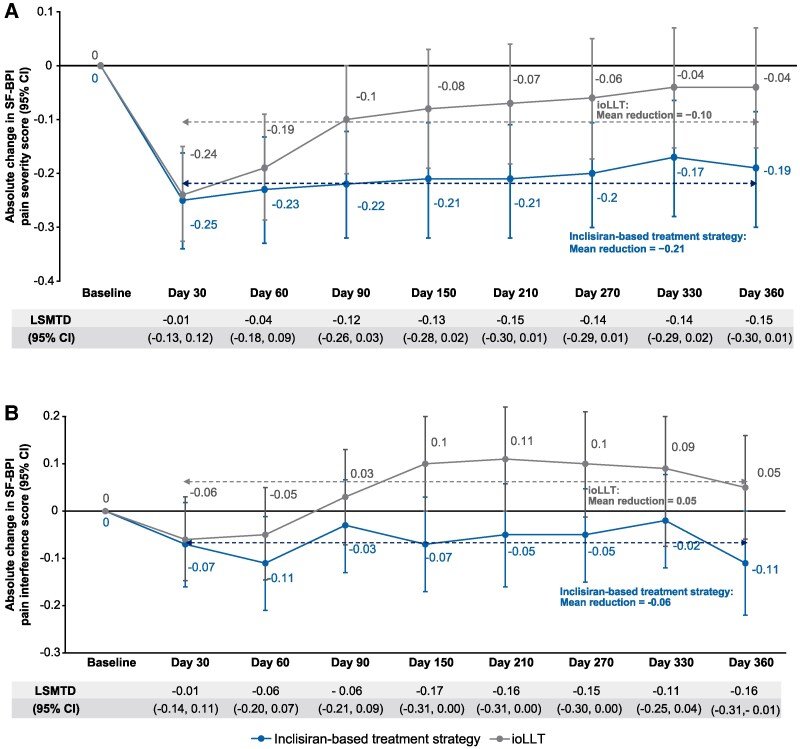
Absolute change in (*A*) SF-BPI pain severity and (*B*) SF-BPI pain interference score from baseline to day 360 in the VICTORION-difference study (full analysis set). The difference in SF-BPI pain severity and interference scores from baseline to Day 360 was not statistically significant. CI, confidence interval; ioLLT, individually optimized LLT; LLT, lipid-lowering therapy; LSMTD, least squares mean treatment difference; SF-BPI, Short-Form Brief Pain Inventory

From baseline to Day 360, significantly fewer participants receiving an inclisiran-based treatment strategy vs ioLLT arm experienced at least one MRAE (11.9% vs 19.2%; OR 0.57, 95% CI: 0.43, 0.74, *P* < .001; *Figure 4*). Additionally, participants receiving an inclisiran-based treatment strategy vs ioLLT also experienced numerically fewer days with pain; however, this difference did not reach statistical significance (198.63 vs 214.51; OR 0.93, 95% CI: 0.81, 1.06, *P* = 0.135; Supplementary data online, *Figure S3*). The change from baseline in SF-BPI pain severity and interference scores favoured inclisiran-based treatment strategy over ioLLT, with between-group differences of −0.11 (95% CI: −0.23, 0.01, *P* = .039) and −0.11 (95% CI: −0.22, 0.00; *P* = .029), respectively (*Figure 5*). Although, treatment with inclisiran vs ioLLT was associated with numerical improvement in SF-BPI pain severity and interference scores as early as Day 60, this difference did not reach statistical significance, with between-group differences of −0.04 (95% CI: −0.18, 0.09) and −0.06 (95% CI: −0.20, 0.07), respectively. However, this difference continued to increase throughout the study duration for SF-BPI pain severity and interference scores to −0.15 and −0.16, respectively, at Day 360.

##### Need for treatment optimization

By Day 330, the proportion of participants in the ioLLT arm who required up-titration of rosuvastatin to MTD, increased substantially as the study progressed, reaching 74.9% compared with only 24.1% among those on an inclisiran-based treatment strategy (see Supplementary data online, *Figure S4*). Notably, as the study progressed, the need for maximizing the statin dose to reach the LDL-C goals was lower in participants receiving inclisiran as the first-choice therapy with statins vs ioLLT (see Supplementary data online, *Figure S5*). At Day 1, although a comparable proportion of participants in both treatment arms required 40 mg rosuvastatin (5.0% and 5.1% in inclisiran and ioLLT arms, respectively), this difference widened in favour of those receiving an inclisiran-based treatment strategy vs ioLLT. By the end of study, only 8.7% of participants receiving an inclisiran-based treatment strategy were on 40 mg rosuvastatin vs 31.4% in the ioLLT arm. Moreover, the need for additional therapy to reach the LDL-C goals was also lower among those receiving inclisiran as first-choice therapy in addition to statins vs ioLLT (see Supplementary data online, *Figure S6*).

### Safety

The overall safety and tolerability profile of an inclisiran-based strategy was comparable to ioLLT (*Table 2*). TEAEs occurring during the study period were reported in 71.3% of participants in the inclisiran arm and 75.9% of participants in the ioLLT arm. Injection-site erythema was reported in 0.1% and 0.0% of participants receiving inclisiran and ioLLT, respectively. Participants experiencing at least one TEAE leading to discontinuation from the double-blind treatment period were also comparable between the two arms (inclisiran, 0.9%; ioLLT, 1.3%).

**Table 2:** Overall summary of TEAEs observed in the VICTORION-difference study (safety set)

	Inclisiran-based treatment strategy *n* = 900	ioLLT *n* = 870
Participants with at least one TEAE	642 (71.3)	660 (75.9)
TEAEs at the maximum severity		
Mild	304 (33.8)	300 (34.5)
Moderate	264 (29.3)	286 (32.9)
Severe	74 (8.2)	74 (8.5)
Participants with at least one TEAE related to double-blind treatment	36 (4.0)	22 (2.5)
Participants with at least one TEAE related to Rosuvastatin	92 (10.2)	165 (19.0)
Participants with at least one TEAE leading to discontinuation from study	3 (0.3)	4 (0.5)
Participants with at least one TEAE leading to discontinuation from double-blind treatment	8 (0.9)	11 (1.3)
Participants with at least one TEAE leading to discontinuation from Rosuvastatin	18 (2.0)	31 (3.6)
Participants with at least one serious TEAE	148 (16.4)	148 (17.0)
Participants who died	6 (0.7)	5 (0.6)

Data presented as *n* (%).

AE, adverse event; ioLLT, individually optimized lipid-lowering therapy; TEAE, treatment emergent AE.

A total of 11 participants across both groups (0.6%) died; the most common cause of death was cardiac disorders, reported in 0.2% and 0.1% of participants receiving inclisiran and ioLLT, respectively (see Supplementary data online, *Table S7*). The incidence of TESAEs was 16.4% and 17.0% in the inclisiran and ioLLT arms, respectively. Across inclisiran and ioLLT treatment arms, the most commonly reported TESAEs were cardiac disorders (5.8% and 3.8%, respectively), followed by musculoskeletal and connective tissue disorders (1.8% and 2.3%, respectively), infections and infestations (2.6% and 2.0%, respectively), vascular disorders (1.4% and 2.1%, respectively), and nervous system disorders (1.4% and 2.1%, respectively; Supplementary data online, *Table S8*). A comparable proportion of participants in both treatment arms experienced new onset of diabetes (inclisiran, 3.0%; ioLLT, 3.3%). No clinically significant changes in laboratory values, vital signs, or ECG parameters were observed.

## Discussion

To our knowledge, VICTORION-Difference is the largest completed LDL-C–lowering study to demonstrate superiority of an inclisiran-based treatment strategy vs placebo, both administered on top of ongoing CV risk-based ioLLT in achieving LDL-C goals as early as Day 90, significantly reducing MRAEs, with reduced need for additional therapies, including up-titration of statin doses. Although previous studies have shown LDL-C reductions from baseline with inclisiran within 14 days^34^ and 90 days of treatment,^29^ findings from the present study demonstrated for the first time the superiority of an inclisiran-based strategy over ioLLT in achieving the primary endpoint of LDL-C goal attainment by Day 90 in the majority of participants with hypercholesterolemia, and high or very high CV risk. Of note, the proportion of participants achieving their individual 2019 ESC/EAS guideline-recommended LDL-C goals after treatment initiation with inclisiran-based treatment strategy vs ioLLT increased by nearly three-fold by Day 90 (84.9% vs 31.0%); leading to 12-× higher odds of achieving treatment goals. Notably, significant LDL-C reductions were observed as early as Day 60 with an inclisiran-based treatment strategy vs ioLLT (57.5% vs 10.9%), which was sustained until Day 360 (−59.7% vs 31.2%); despite receiving individualized treatment, reductions in the ioLLT arm remained consistently lower (*Structured Graphical Abstract*).

The superior clinical efficacy of inclisiran was also accompanied by significantly fewer participants experiencing an MRAE vs ioLLT (11.9% vs 19.2%), resulting in 57% increased rate of MRAEs among those not treated with an inclisiran-based strategy. Participants treated with an inclisiran-based strategy also reported numerical improvement, though not statistically significant, in pain-related QoL, with change from baseline in SF-BPI pain severity and interference scores favouring inclisiran vs ioLLT (−0.11, *P* = 0.039; and −0.11, *P* = .029, respectively). Statin-related muscle pain continues to remain an ongoing challenge for managing ASCVD,^18^ which hinders the achievement of recommended LDL-C goals, thereby increasing the risk of recurrent CV events. Consequently, in patients with statin intolerance, early intensification of drug therapy is recommended by combining LLTs with a low-dose statin to help achieve LDL-C goals and reduce the risk of recurrent CV events.^12,35^ Findings from the VICTORION-Difference study demonstrated that using inclisiran as the first-choice therapy in addition to statins enabled LDL-C goal attainment earlier than ioLLT in most at-risk participants, without the need for up-titrating rosuvastatin dose or additional therapies. In contrast, most participants not receiving inclisiran as part of individualized LLT, including statin MTD and additional therapies, failed to achieve their LDL-C goals and experienced MRAEs, highlighting the need for effective lipid-lowering approaches. Furthermore, the convenient dosing schedule of inclisiran, combined with likelihood of reduced MRAEs has the potential to improve adherence of individuals who fail to achieve their LDL-C goals with MTD of statins. This is of significant clinical importance since prompt initiation of a LLT that enables early LDL-C goal attainment without the need for escalating statin doses or additional therapy, thereby potentially simplifying treatment regimen, minimizing MRAEs and improving long-term adherence, particularly for those at high or very high CV risk.^36^  ^,37^ Indeed, results of a recent meta-analysis of 14 studies including over 100 000 very high-risk patients showed that combination LLT was associated with an overall greater reduction in LDL-C and significantly lower risk of all-cause mortality, major adverse CV events, and stroke compared with statin monotherapy.^36^ Furthermore, findings of the present trial strongly reinforce and align with the results of an observational study of over 56 000 patients from the SWEDEHEART registry, which demonstrated that an early and sustained reduction in cholesterol levels after a myocardial infarction is linked to a lower risk of major adverse CV events.^38^ This challenges the conventional stepwise approach, which delays the initiation of combination LLT. Such delays, due to lack of appropriate treatment escalation strategies, can lead to potentially preventable harm to patients.^38,39^ Therefore, using an inclisiran-based treatment strategy vs ioLLT provides a promising therapeutic approach to bring more at-risk patients to early (Day 90) and sustained LDL-C goal attainment in the majority of participants with hypercholesterolemia, together with lower incidence of MRAEs. Interestingly, participants in both arms experienced an improvement in pain-related QoL when initially switching to rosuvastatin; however, this QoL improvement was better sustained with an inclisiran-based treatment strategy vs ioLLT. Although the difference was evident as early as Day 60 and persisted throughout the study, it did not reach statistical significance in the overall population, likely due to a larger potential impact in a minority of the participants experiencing QoL impairment from MTD of statin therapy. The present study also confirmed that the safety and tolerability profile of an inclisiran-based strategy was comparable to ioLLT. The overall safety profile remained favourable and consistent with what was previously reported,^29,30^ with no new safety concerns identified.

### Study limitations

The relatively short duration of the study and follow-up period may have precluded an assessment of the long-term benefits of inclisiran. Since all study participants were recruited from European countries (96.9% white) and 69.8% were males, outcomes may have been skewed toward the experiences and responses of the overrepresented group, potentially limiting generalizability to more diverse populations. Although this was a randomized controlled trial with respect to the administration of inclisiran and placebo, the administration of open-label background rosuvastatin varied per individual patient needs. Since the majority of study participants were classified as having very high CV risk, close monitoring of LDL-C levels was considered important. Therefore, LDL-C levels were measured at each visit using a point-of-care device, allowing investigators to make immediate decisions on LLT adjustments, while LDL-C levels for endpoint analysis, were assessed centrally and blinded. The study protocol specified that the rosuvastatin dose would not be blinded between the two treatment arms to enable ongoing individual LLT optimization at investigator’s discretion, to better reflect real-world clinical practice. The randomized, double-blind, placebo-controlled design of the trial effectively minimized bias by limiting confounding factors, such as investigators’ access to LDL-C measurements, which guided treatment intensification decisions related to use of open-label rosuvastatin therapy, including dose escalation or the addition of other LLTs over time. The study design also allowed for the administration of anti-PCSK9 mAb or local ioLLT after discontinuing inclisiran/placebo treatment (after the double-blind treatment period). While the present trial did not evaluate the impact of an inclisiran-based treatment strategy on mortality, it is noteworthy that existing data from other studies suggest a reduction in a composite endpoint of myocardial infarction, CV mortality, and stroke in patients with established ASCVD.^40,41^ Additionally, this study did not compare the impact of an inclisiran-based treatment strategy vs ioLLT on health economic outcomes, which likely may vary across countries and healthcare systems; further research is needed to explore these aspects, potentially incorporating digital tools to support personalized care and improve adherence. Altogether, VICTORION-Difference is the largest LDL-C–lowering study with 1770 participants, and the first to evaluate the effect of an inclisiran-based treatment strategy vs ioLLT on patient-reported outcomes, including the novel endpoint of MRAEs and pain-related QoL scores as secondary endpoints. A key strength of this clinical study is that the trial design closely mirrors patient-centric real-world care. With over 90% of participants at very high CV risk, the study reflects real-world populations where suboptimal LDL-C levels are common,^13–15^ providing insight into inclisiran’s efficacy and safety in those most vulnerable to recurrent CV events and enhancing the clinical relevance of the findings. Another key feature of the present study is that it is the first to evaluate the effect of inclisiran vs placebo on top of ioLLT on impairment due to statin-related muscle pain; this was achieved using patient-centric QoL measures, such as the SF-BPI and pain diary, as secondary endpoints. These results provide a comprehensive view of how treatment with inclisiran impacted patients’ daily lives, beyond lowering LDL-C levels. While the SF-BPI offered insights into pain severity and its interference with daily activities, the pain diary captured multidimensional experience of recurrent pain in real-time.^42^ Understanding patient perspectives is key to improving outcomes, yet standardized tools to capture these are rarely used in trials; their integration in the VICTORION-Difference study enabled a more accurate evaluation of efficacy and safety of an inclisiran-based treatment strategy, thereby aligning with patient-centred care.^43^

## Conclusion

The VICTORION-Difference study met its primary objective showing that, in participants with hypercholesterolaemia on MTD of statins, treatment with an inclisiran-based treatment strategy was superior to placebo with ioLLT in attainment of LDL-C goals by Day 90. Additionally, treatment with an inclisiran-based strategy vs ioLLT was associated with significant LDL-C reduction, as early as Day 60 and was sustained over the trial period. Using patient-centric QoL measures, this study demonstrated for the first time that participants treated with background rosuvastatin experienced significantly lower MRAEs and numerical reduction in pain-related QoL when receiving inclisiran-based treatment strategy vs ioLLT. Findings of the study demonstrated that an inclisiran-based treatment strategy had a comparable safety and tolerability profile to ioLLT, with no new safety findings. Overall, these results support the therapeutic benefit of an inclisiran-based treatment strategy in bringing more participants with hypercholesterolaemia at high and very high CV risk to their LDL-C goal attainment by Day 90, while numerically improving QoL measures without the need for additional therapy or up-titration of statin dose.

## Supplementary Material

ehaf685_Supplementary_Data

## Declarations

### Data Availability

Qualified researchers can request access to patient-level data and related study documents after publication, including the statistical analysis plan and dataset specifications. Patient-level data was anonymized, and study documents were redacted to protect the privacy of the trial participants.

### Pre-registered Clinical Trial Number

The pre-registered clinical trial number is NCT05192941.
