# Supplementary material for: Inclisiran-based treatment strategy in hypercholesterolaemia: the VICTORION-difference trial
Source: Eur Heart J. 2025 Aug 30;47(25):3241–53. doi: 10.1093/eurheartj/ehaf685 (PMC13318417; doi:10.1093/eurheartj/ehaf685)
Supplement: ehaf685_Supplementary_Data [file ehaf685_supplementary_data.docx]

**Inclisiran-based treatment strategy in hypercholesterolaemia: the VICTORION-Difference trial**

**Authors:**

Ulf Landmesser (MD)^1,2,*^, Ulrich Laufs (MD)^3^, Ulrike Schatz (MD)^4^; Ephraim B. Winzer (MD)^5^; Bernd Nowak (MD)^6^; Ursula Kassner (MD)^7^; Ioanna Gouni-Berthold (MD)^8^; Alicia Esteban (PhD)^9^; Lawrence Lubyayi (PhD)^10^; Andre Krueger (MSc)^11^; Christian Hentschke (MSc)^12^; Andreas Wilke (MD)^13^; Bernhard R. Winkelmann (MD)^14^; Assya Achouba (MD)^15^; Maciej Banach (MD PhD)^16,17,18^

**Affiliations:**

^1^Deutsches Herzzentrum der Charité, Klinik für Kardiologie, Angiologie und Intensivmedizin, Hindenburgdamm 30, 12203 Berlin, Germany; ^2^Charité—Universitätsmedizin Berlin, corporate member of Freie Universität Berlin and Humboldt-Universität zu Berlin, Charitéplatz 1, 10117 Berlin, Germany; ^3^Department of Cardiology, Klinik und Poliklinik für Kardiologie, Universitätsklinikum Leipzig, Leipzig, Germany; ^4^Department of Internal Medicine III, Faculty of Medicine at the Technical University of Dresden, University Hospital Carl Gustav Carus, Dresden, Germany; ^5^Department for Internal Medicine and Cardiology, Heart Center Dresden, University Clinic, Technische Universität Dresden, Dresden, Germany; ^6^CCB, Cardioangiologisches Centrum Bethanien, Frankfurt a.M., Germany; ^7^Division of Lipid Disorders, Department of Endocrinology, Charité–Universitätsmedizin Berlin, corporate Member of Freie Universität Berlin and Humboldt-Universität zu Berlin, Berlin, Germany; ^8^Center for Endocrinology, Diabetes and Preventive Medicine, Faculty of Medicine and University Hospital Cologne, University of Cologne, Cologne, Germany; ^9^Clinical Development, Novartis Pharma AG, Basel, Switzerland; ^10^Advanced Quantitative Sciences, Novartis Pharmaceuticals UK Ltd, London, UK; ^11^Global Medical Affairs, Cardio Renal Metabolism, Novartis Pharma AG, Basel, Switzerland; ^12^Evidence Generation, Novartis Pharma GmbH, Nürnberg, Bayern, Germany; ^13^Kardiologische Praxis Papenburg, Papenburg, Germany; ^14^Study Center, ClinPhenomics CVC GmbH, Frankfurt, Germany; ^15^Global Medical Affairs, ASCVD, Novartis Pharma AG, Basel, Switzerland; ^16^Faculty of Medicine, the John Paul II Catholic University of Lublin, Lublin, Poland; ^17^Ciccarone Center for the Prevention of Cardiovascular Disease, Johns Hopkins University School of Medicine, Baltimore, MD, USA; and ^18^Department of Preventive Cardiology and Lipidology, Medical University of Lodz, Lodz, Poland

*Corresponding author. Email: [ulf.landmesser@dhzc-charite.de](mailto:ulf.landmesser@dhzc-charite.de)

# **SUPPLEMENTARY METHODS**

**Trial Design**

The overall study duration was ≥360 days and included: (i) a screening period of ~14 days for all participants; (ii) an optional run-in period of ≤120 days as individually applicable; (iii) an optional additional baseline period of ~10 days as individually applicable for required labs; and a treatment period of 360 days (excluding a safety follow-up call 30 days after the end of study).

All participants or their legal representative(s) provided signed informed consent in accordance with regulations set by the local ethics review committee.

The study was conducted in accordance with the study protocol, the International Conference on Harmonization guidelines, the Declaration of Helsinki, and local ethics committee approvals. The study was approved by the ethics committee and institutional review board of each participating country.

## **Trial population**

Key exclusion criteria were described previously^1^ and included, among others, presence of severe concomitant non-CV disease expected to reduce life expectancy to less than 2 years at screening or baseline visit, receiving ≥1 LLT on top of statin at screening visit, and secondary hypercholesterolaemia, e.g. hypothyroidism or nephrotic syndrome at screening or baseline visit.

**Statistical analysis**

For the primary and secondary efficacy endpoints, missing values were handled using multiple imputation. Multiple imputation was used following the steps outlined below:

For missing data, the target day of the visit was considered to assess if missingness was related or not related to an intercurrent event. Absolute values were imputed and corresponding endpoint variables were derived based on imputed data. Data were selected to fit the imputation model considering intercurrent events strategies as below:

**Step 1:** For the primary endpoint all observed data were included except for data collected after intercurrent events where the composite strategy was applied, e.g. data collected after use of PCSK-9 inhibitors were set to missing. Note, after death no data are collected, thus no need to take any action for the while alive strategy. In case of occurrence of multiple intercurrent events e.g. double-blind treatment discontinuation and later anti-PCSK-9 mAb use, all retrieved dropout (RDO) data up to use of PCSK-9 inhibitors were used, others were set to missing.

**Step 2:** For secondary endpoints, all observed data were included except for data collected after intercurrent events where the hypothetical strategy was applied, these were set to missing after intercurrent events. In case of occurrence of multiple intercurrent events e.g. double-blind treatment discontinuation and later anti-PCSK-9 mAb use, all RDO data up to PCSK-9 inhibitor use will be used, others were set to missing for LDL-C analyses where hypothetical strategy is applied for PCSK-9 inhibitor use.

**Step 3:** Impute missing data using MAR (missing at random): Select all participants, impute missing values at baseline and scheduled post-baseline visits, under assumption of missing at random (MAR), using the PROC MI procedure in SAS based on the fully conditional specification (FCS statement) method for 100 times and obtain 100 imputed datasets. Missing baseline values will be imputed using a model with treatment and CV risk category. Missing Day 30 values would be imputed using a model based on values of baseline, treatment and CV risk category. The same procedure would be repeated for subsequent visits.

This results in 100 imputed datasets.

For participants in the inclisiran treatment group who discontinued double-blind treatment prematurely, set imputed values after discontinuation of double-blind treatment to missing, if not after occurrence of other intercurrent events such as PCSK-9 inhibitor use, as these would be imputed using jump to reference (J2R) in step 4.

**Step 4:** For intercurrent event of double-blind treatment discontinuation, in case of missing RDO data and no occurrence of other intercurrent event such as anti-PCSK-9 mAb use, impute missing values for participants in the inclisiran treatment groups using J2R.

Select all participants, impute missing values at scheduled post-baseline visits using the MI approach, under assumption of missing not at random (MNAR) that participants in inclisiran treatment groups will behave as participants treated with placebo, based on the fully conditional specification (FCS) method for 100 times and obtain 100 imputed datasets. Model will include same covariates as in step 1, except for treatment which is considered in the MNAR assumption.

This results in 100 imputed datasets.

**Step 5:** Merge datasets from steps 3 and 4.

- 1. For participants in the inclisiran treatment groups discontinuing double-blind treatment prematurely take imputed values after discontinuation of double-blind treatment from step 4 if not after occurrence of other intercurrent event such as PCSK-9 inhibitor.
  2. For all other missing (including those for placebo participants) values take imputed values from step 3.

**Step 6:** Derive endpoint variable for imputed values, e.g. change from baseline/response variable/percent change from baseline.

**Step 7:** For each considered endpoint the specified analysis model would be applied on each of the 100 final multiply-imputed datasets where all missing values are filled (from MI or single imputation as defined by the respective strategy).

The results for the treatment effects from the 100 datasets were then combined to obtain the statistical quantities of interest using Rubin's rule. Therefore, in SAS the PROC MIANALYZE procedure was used and generates valid statistical inferences. If models failed to converge for a subset of imputations the remaining ones were used.

# **SUPPLEMENTARY TABLES**

**Table S1. The standardised form utilised by the board of independent experts to review requests for PCSK9 mAb during the VICTORION-Difference study**

| CKJX839A12402 (Victorion Difference): Request Form – PCSK9 mAb Eligibility Review | | | |
| --- | --- | --- | --- |
| Section to be completed by Investigator | | | |
| Investigator Name: | |  | |
| Site Number: | |  | |
| Subject Number: | |  | |
| Date of Request (DD-MMM-YYYY) | |  | |
| CV risk category assessment (please provide details of cardiovascular disease history).  **Notes**:   1. *If patient has ASCVD confirmed upon imaging, please provide detailed description of finding (e.g. location and degree of stenosis) and imaging technique used* 2. *If patient has diagnosed Familial Hypercholesterolemia please provide details on:*  - *Diagnostic criteria used (e.g. genetic testing, dutch lipid clinic score etc)* - *Age of index CV event (if applicable)* - *Family CVD history (if known)* | |  | |
| **Current rosuvastatin dose:**  **Note:** *If current dose is <40 mg, please provide detailed history of prior statin use, and rationale why lower dose (<40 mg) is considered the max tolerated dose* | |  | |
| **Currently receiving Ezetimibe**  *If “No”*  Please confirm if patient previously received Ezetimibe  If patient is not currently on Ezetimibe, please provide reason: | | YES  NO  YES  NO | |
| Currently receiving Bile acid sequestrant  *If “No”*  Please confirm if patient previously received Bile acid sequestrants  If patient is not currently on a bile acid sequestrant please provide reason: | | YES  NO  YES  NO | |
| Currently receiving Bempedoic Acid  *If “No”*  Please confirm if patient previously received Bempedoic Acid  If patient is not currently on Bempedoic Acid please provide reason: | | YES  NO  YES  NO | |
| Any other relevant information | |  | |
| Investigator signature | |  | |
| Section to be completed by PCSK9 mAb Review board | | | |
| Request Reviewed by: | **Prof. Dr, med. Bernd Nowak:** | | **Approved**:  **Denied**: |
|  | Signature: | |  |
|  | Date of Review: | |  |
|  | **Dr, med. Ursula Kassner** | | **Approved**:  **Denied**: |
|  | Signature: | |  |
|  | Date of Review: | |  |
|  | **Dr. med. Ephraim Winzer** | | **Approved**:  **Denied**: |
|  | Signature: | |  |
|  | Date of Review: | |  |
| Overall Request Status | **Approved**: | | **Denied**: |
| If Request is Denied, please provide supporting rational |  | | |
| Additional Comments |  | | |

**ASCVD atherosclerotic cardiovascular disease; CV, cardiovascular; mAb, monoclonal antibody; PCSK9, proprotein convertase subtilisin/kexin type 9.**

**Table S2. PRO measures assessed in the VICTORION-Difference study**

| **SF-BPI** | **The SF-BPI is a self-administered standardised fifteen items questionnaire that assesses how pain interferes with or influences a participant’s life. The query period covers the past 24 hours and takes 5 minutes for the participant to complete. The first item is a screening question about the participant's pain on the day. The questionnaire is then composed of pain drawing diagrams, four items about pain intensity (worst pain, least pain, average pain, pain right now), two items on pain relief treatment or medication, and one item on pain interference, with seven sub-items (general activity, mood, walking ability, normal walk, relations with other people, sleep, and enjoyment of life). The SF-BPI includes two main scores: a pain severity score and a pain interference score. The pain severity score combines the information of the four items about pain intensity, which are rated from 0, no pain, to 10, pain as bad as you can imagine. To derive the pain severity score the average of the four items will be taken The pain interference is calculated similarly using the seven items regarding pain interference, which are rated from 0, does not interfere, to 10, completely interferes. The pain interference score was taken as the average of these seven items. Both scores were between 0 and 10. The remaining items if the questionnaire do not contribute to the scoring. Higher scores correspond to a poorer condition of the participant. The first item, pain drawing diagrams (painful and most painful areas) and the items on pain relief treatment or medication (list of the treatments and amount of relief) do not contribute to the scoring. It has been validated^2,3^ for use with non-malignant pain populations and has been used in statin trials.^4^** |
| --- | --- |
| **Pain Diary** | **The Pain Diary is based on item-3 of SF-BPI. Daily pain was rated on a numeric scale from 0 (no pain) to 10 (pain as bad as you can imagine) by the participants describing their pain at its worst in the last 24 hours. All participants completed the diary via a handheld an electronic device. Investigators instructed the participants to fill in the diary on a daily basis and to adhere to a certain routine for completing the diary** |
| **SF-36 V. 2** | **The SF-36 is a generic HRQoL instrument which comprises of 36 questions across 8 domains: 1) limitations in physical activities because of health problems; 2) limitations in social activities because of physical or emotional problems; 3) limitations in usual role activities because of physical health problems; 4) bodily pain; 5) general mental health (psychological distress and well-being); 6) limitations in usual role activities because of emotional problems; 7) vitality (energy and fatigue); 8) general health perceptions.^5^ Two overall summary scores, the PCS and the MCS were computed.^6^ An increase in the scale values reflects an improvement in the quality of life.^5^ The SF-36 is considered to be a valid, reliable, concise generic measure of state of health and has demonstrated to detect clinical treatment benefits across medical conditions including chronic disorders such as dyslipidaemia.^7^ The SF-36 is regularly used as a generic questionnaire in clinical studies in the field of cardiology^8^ and is the most frequently used generic questionnaire in clinical studies with PAD participants.^9^ In the cardiovascular participant population, especially those with CHD, it is considered to be the most reliable and change-sensitive generic questionnaire.^10^ The SF-36 has been validated at the international level for the chronically ill, including cardiac participants.^11,12^ In addition, the HRQoL project resulted in international reference values for the SF-36 for heart participants.^13^** |
| **GSRS** | The self-administered GSRS questionnaire contains 15 items for gastrointestinal syndromes, which are composed of 5 dimensions (abdominal pain, reflux syndrome, indigestion, diarrhoea and constipation) and use a Likert scale with 7 units (1 being no discomfort to 7 being very  severe discomfort).^14-16^ The sub-scores of individual domains are calculated by averaging the scores of the associated items. The average of all sub-scores gives the total score, which ranges from 1 to 7 points. The lower the value, the better the gastrointestinal condition. The reliability and validity of the GSRS are well documented,^17-19^ and norm values for a general population are available.^16,20^ The GSRS has been taken isolated into account in the present indication^19^ and therapeutic area of "metabolic diseases". |
| **TSQM v. 2** | TSQM is a validated participant reported outcome instrument^21,22^used to assess participant satisfaction with treatment. All subjects were provided with the TSQM v. II, an 11-item multiple choice questionnaire validated to assess convenience and global satisfaction with treatment. TSQM is a widely used generic measure, including dyslipidemia,^23,24^ to assess the major dimensions of patients’ satisfaction with medication and has been psychometrically validated in a heterogeneous sample.^21,22^ TSQM v. II is comprised of 11 questions that provide scores on four scales: effectiveness (2 items), side effects (4 items), convenience (3 items) and global satisfaction (2 items) over the previous 2–3 weeks, or since the participant’s last use. With the exception of item 4 (presence of side effects; yes or no), all items have five or seven responses, scored from one (least satisfied) to five or seven (most satisfied). Item scores are summed to give four domain scores, which are in turn transformed to a scale of 0–100. The higher the value, the higher is the participant’s satisfaction with the treatment. |
| **WIQ** | The WIQ measures self-reported walking distance, walking speed, and stair-climbing ability in men and women with lower extremity PAD.^25^ The modified WIQ records a total of 16 items in four categories: pain (2 questions), walking distance (7 questions), walking speed (4 questions), and stair climbing (3 questions). In the WIQ distance score, the participant records the degree of difficulty walking specific distances (ranging from walking indoors to 1,500 feet or 5 blocks) on a graded Likert- scale from 0 to 4. A score of 0 represents the inability to walk the distance in question and a score of 4 represents no difficulty. In the WIQ speed score, the participant is asked to assess the degree of difficulty walking 1 block at specific speeds ranging from walking slowly to jogging on a graded scale ranging from 0 to 4. In the WIQ stair climbing score, the participant reports the degree of difficulty climbing 1, 2, and 3 flights of stairs. This graded score is multiplied by a pre-specified weight for each distance, speed, or number of stair flights. The products are summed and divided by the maximum possible score to obtain a percent score, ranging from 0 (representing the inability to perform any of the tasks) to 100 (representing no difficult with any of the tasks)^26^ The WIQ scores have been shown to improve in response to lower extremity revascularization^27^ and supervised exercise therapy.^28^ The WIQ has been validated several times in PAD participants^27,29-31^ and also provides good predictions of cardiovascular risk in other cardiovascular diseases.^32^ The WIQ was only completed by participants diagnosed with PAD at baseline. |
| **MacNew Heart Disease Quality of Life Questionnaire (MacNew)** | The MacNew Heart Disease HRQoL questionnaire (Mac-New) is a self-administered modification of the original QLMI instrument.^33,34^ It was specially developed to measure the quality of life of participants with cardiovascular diseases. The MacNew consists of 27 items which fall into three domains (physical limitations, emotional and social function domain scale). There are 5 items that inquire about symptoms: angina/chest pain, shortness of breath, fatigue, dizziness, and aching legs. The participants answer the questions using a seven-point Likert scale from 1 ("worst feeling") to 7 ("best feeling"). The scores of the three individual domains are calculated by averaging the point values in the associated items. The average value of all 27 items gives the global total score. A higher total score corresponds to a better quality of life. The validity, reliability and change sensitivity of the MacNew have been demonstrated in various participant groups, including participants with myocardial infarction, with AP and with ischemic heart failure.^35,36^ The Mac New questionnaire was only completed by participants diagnosed with angina pectoris at baseline. The query period covered the past 14 days. |

**CHD, coronary heart disease; GSRS, Gastrointestinal Symptom Rating Scale; HRQoL, health-related quality of life; MCS, mental component summary; PAD, peripheral arterial disease; PCS, physical component summary; PRO, patient reported outcome; QLMI,** Quality of Life after Myocardial Infarction; **SF-36, Short-Form Health Survey; SF-BPI,** Short-Form Brief Pain Inventory; TSQM, Treatment Satisfaction Questionnaire for Medication; WIQ, Walking Impairment Questionnaire.

**Table S3. Participant disposition based on inclusion criteria not met for participants who did not go through the run-in period (n=544)**

| **Inclusion criteria not met for participants who did not go through the run-in period (Screened set)** | |
| --- | --- |
|  | **Total     N=2529     n (%)** |
| Discontinued prior to randomization* | 544 |
|  | |
| Inclusion criterion not met | |
| LDL-C levels according to their risk level at screening | 360 (66.2) |
| Fasting triglyceride <400 mg/dL at screening. | 37 (6.8) |
| Participants meeting the CV categories of very high or high risk as defined in 2019 ESC/EAS guidelines | 8 (1.5) |
| Participants on a stable dose of a statin for ≥30 days | 8 (1.5) |
| LDL-C levels according to their risk level at baseline | 5 (0.9) |
| Written informed consent obtained before any assessment is performed | 3 (0.6) |
| Male or female participants ≥18 years of age | 1 (0.2) |
| Stable dose (≥30 days prior to screening) of another LLT on top of statin (up to approximately 20% of total participants) | 1 (0.2) |
|  |  |
| Exclusion criterion met |  |
| Unwillingness or inability to comply with study procedures, and medication administration and schedule | 68 (12.5) |
| Participant with severe renal impairment defined by eGFR <30 mL/min/1.73m^2^ as calculated by the Modification in Diet in Renal Disease (MDRD) formula at screening or baseline visit | 11 (2.0) |
| Secondary hypercholesterolemia, e.g. hypothyroidism or nephrotic syndrome at screening or baseline visit | 7 (1.3) |
| Liver and CK: (a) Active liver disease or (b) ALT, AST >3x ULN, or TBIL > 2x ULN, or (c) CK >5x ULN at screening or baseline | 6 (1.1) |
| Previous, current or planned treatment with LDL-apheresis at screening or baseline visit | 5 (0.9) |
| Participants on more than one other lipid-lowering drug on top of statin at screening visit | 4 (0.7) |
| Acute CS, IS or TIA, CR or PA revascularization procedure or amputation due to ASD <3 months prior to the screening or baseline | 4 (0.7) |
| Any surgical or medical condition, which in the opinion of the investigator, may place the participant at higher risk from his/her participation in the study | 4 (0.7) |
| History of malignancy that required surgery, radiation therapy and/or systemic therapy during 3 years prior to screening or baseline | 3 (0.6) |
| Severe concomitant non-CV disease that is expected to reduce life expectancy to less than 2 years at screening or baseline visit | 2 (0.4) |
| Participants with known intolerance to rosuvastatin at screening or baseline visit | 2 (0.4) |
| Women of child-bearing potential | 2 (0.4) |
| Pre-existing diagnosis of homozygous familial hypercholesterolemia at screening or baseline visit | 1 (0.2) |
| Previous exposure to inclisiran or any other non-mAb PCSK9 targeted therapy, either as an investigational or marketed drug within 2 years prior to screening or baseline visit | 1 (0.2) |
| Planned or expected cardiac, cerebrovascular or peripheral artery surgery or coronary revascularization within the study duration | 1 (0.2) |
| Heart failure NYHA class IV at screening or baseline visit | 1 (0.2) |
| Use of other investigational drugs within 5 half-lives, 30 days or until the expected pharmacodynamic effect has returned to baseline, prior to screening visit | 1 (0.2) |

*Including mis-randomized participants. Mis-randomized participants are participants randomised by mistake who were subsequently deemed screen failures and did not receive study treatment.

N, number of participants screened. Percentages are based on the number of participants discontinued prior to randomization.

The screened set consisted of all participants who signed the informed consent. The screened set included only unique screened participants, i.e., in the case of re-screened participants only the chronologically last screening data was counted.

ASD, atherosclerotic disease; CR, coronary revascularization; CS, coronary syndrome; CV, cardiovascular; ESC, European Society of Cardiology; EAS, European Atherosclerosis Society; IS, ischemic stroke; LDL-C, low-density lipoprotein cholesterol; LLT, lipid-lowering therapy; mAb, monoclonal antibody; NYHA, New York Heart Association; PA, peripheral arterial; PCSK9, proprotein convertase subtilisin/kexin type 9; TBIL, total bilirubin; TIA, transient ischaemic attack; ULN, upper limit of normal.

**Table S4. Participant disposition based on inclusion criteria not met for participants who went through the run-in period (n=215)**

| **Inclusion criteria not met for participants who went through the run-in period (Run-in set)** | |
| --- | --- |
|  | **Total     N=696     n (%)** |
| Discontinued prior to randomization* | 215 |
|  |  |
| Inclusion criteria not met |  |
| LDL-C levels according to their risk level at baseline | 86 (40.0) |
| LDL-C levels according to their risk level at screening | 58 (27.0) |
| Fasting triglyceride <400 mg/dL at baseline | 6 (2.8) |
| Fasting triglyceride <400 mg/dL at screening | 4 (1.9) |
|  |  |
| Exclusion criteria met |  |
| Unwillingness or inability to comply with study procedures, and medication administration and schedule | 29 (13.5) |
| Secondary hypercholesterolemia, e.g. hypothyroidism or nephrotic syndrome at screening or baseline visit | 7 (3.3) |
| Participant with severe renal impairment defined by eGFR <30 mL/min/1.73m^2^ as calculated by the Modification in Diet in Renal Disease (MDRD) formula at screening or baseline visit | 5 (2.3) |
| History of hypersensitivity to any of the study treatments, inclisiran or rosuvastatin, or its excipients or to drugs of similar chemical classes at screening or baseline visit | 4 (1.9) |
| Acute CS, IS or TIA, CR or PA revascularization procedure or amputation due to ASD <3 months prior to the screening or baseline. | 3 (1.4) |
| Participants with known intolerance to rosuvastatin at screening or baseline visit | 2 (0.9) |
| Liver and CK: (a) Active liver disease or (b) ALT, AST >3x ULN, or TBIL > 2x ULN, or (c) CK >5x ULN at screening or baseline | 2 (0.9) |
| Planned or expected cardiac, cerebrovascular or peripheral artery surgery or coronary revascularization within the study duration | 2 (0.9) |
| Participants on more than one other lipid-lowering drug on top of statin at screening visit | 1 (0.5) |
| History of malignancy that required surgery, radiation therapy and/or systemic therapy during 3 years prior to screening or baseline | 1 (0.5) |
| Any surgical or medical condition, which in the opinion of the investigator, may place the participant at higher risk from his/her participation in the study | 1 (0.5) |

*Including mis-randomized participants. Mis-randomized participants are participants randomised by mistake who were subsequently deemed screen failures and did not receive study treatment.

N, number of participants screened. Percentages are based on the number of participants discontinued prior to randomization.

The run-in set consisted of all patients who enter the run-in phase.

ASD, atherosclerotic disease; CK, creatine kinase; CR, coronary revascularization; CS, coronary syndrome; CV, cardiovascular; EAS, European Atherosclerosis Society; eGFR, estimated glomerular filtration rate ESC, European Society of Cardiology; IS, ischemic stroke; LDL-C, low-density lipoprotein cholesterol; LLT, lipid-lowering therapy; mAb, monoclonal antibody; PA, peripheral arterial; TBIL, total bilirubin; TIA, transient ischaemic attack; ULN, upper limit of normal.

**Table S5. Additional baseline characteristics of participants included in the VICTORION-Difference study (full analysis set)**

| **Characteristic  Statistic/category** | **Inclisiran-based treatment strategy N=898** | **ioLLT N=872** | **Total N=1770** |
| --- | --- | --- | --- |
| Creatinine clearance* (mL/min) |  |  |  |
| N | 474 | 441 | 915 |
| Median | 96.0 | 94.0 | 95.0 |
| Q1–Q3 | 76.0–123.0 | 73.0–122.0 | 74.0–122.0 |
| eGFR (mL/min/1.73m^2^) |  |  |  |
| N | 898 | 872 | 1770 |
| Median | 80.0 | 79.0 | 80.0 |
| Q1–Q3 | 67–92 | 66–94 | 66–93 |
| Fasting glucose (mmol/L) |  |  |  |
| N | 895 | 865 | 1760 |
| Median | 6.1 | 6.0 | 6.0 |
| Q1–Q3 | 5.5–7.3 | 5.6–7.3 | 5.5–7.3 |
| HbA1c (%) |  |  |  |
| N | 897 | 871 | 1768 |
| Median | 5.9 | 5.9 | 5.9 |
| Q1–Q3 | 5.6–6.4 | 5.6–6.5 | 5.6–6.5 |
| Lp(a) (mg/dL) |  |  |  |
| N | 894 | 867 | 1761 |
| Median | 16.30 | 16.60 | 16.60 |
| Q1–Q3 | 6.0–68.9 | 6.0069.9 | 6.069.2 |
| AST (U/L) |  |  |  |
| N | 898 | 872 | 1770 |
| Median | 17.0 | 17.0 | 17.0 |
| Q1–Q3 | 14.0–21.0 | 14.0–21.0 | 14.0–21.0 |
| ALT (U/L) |  |  |  |
| N | 898 | 872 | 1770 |
| Median | 20.0 | 20.0 | 20.0 |
| Q1–Q3 | 15.0–27.0 | 15.0–27.0 | 15.0–27.0 |
| Total bilirubin (µmol/L) |  |  |  |
| N | 898 | 872 | 1770 |
| Median | 10.4 | 10.6 | 10.6 |
| Q1–Q3 | 8.2–13.3 | 8.4–13.8 | 8.4–13.5 |

*Creatinine clearance was only measured for a subset of patients as this assessment was added in the second amendment of the protocol.

ALT, alanine transaminase; AST, aspartate aminotransferase; eGFR, estimated glomerular filtration rate; ioLLT, individually optimized lipid-lowering therapy; Lp(a), Lipoprotein(a); Q, quartile.

**Table S6. Countries of participants included in the VICTORION-Difference study (full analysis set)**

| **Characteristic  Statistic/category** | **Inclisiran-based treatment strategy N=898** | **ioLLT**  **N=872** | **Total N=1770** |
| --- | --- | --- | --- |
| Country, n (%) | | | |
| Bulgaria | 98 (10.9) | 103 (11.8) | 201 (11.4) |
| Czech Republic | 62 (6.9) | 59 (6.8) | 121 (6.8) |
| Estonia | 46 (5.1) | 48 (5.5) | 94 (5.3) |
| France | 24 (2.7) | 19 (2.2) | 43 (2.4) |
| Germany | 501 (55.8) | 481 (55.2) | 982 (55.5) |
| Latvia | 31 (3.5) | 29 (3.3) | 60 (3.4) |
| Poland | 80 (8.9) | 78 (8.9) | 158 (8.9) |
| Spain | 56 (6.2) | 55 (6.3) | 111 (6.3) |

**ioLLT, individually optimized lipid-lowering therapy**.

**Table S7. Deaths reported during the VICTORION-Difference study by primary system organ class and preferred term (safety set)**

| **Primary system organ class     Preferred term** | **Inclisiran-based treatment strategy N=900 n (%)** | **ioLLT N=870 n (%)** |
| --- | --- | --- |
| Any primary system organ class | 6 (0.7) | 5 (0.6) |
| Cardiac disorders | 2 (0.2) | 1 (0.1) |
| Cardiogenic shock | 1 (0.1) | 0 |
| Coronary artery insufficiency | 1 (0.1) | 0 |
| Myocardial infarction | 0 | 1 (0.1) |
| General disorders and administration site conditions | 0 | 3 (0.3) |
| Death | 0 | 2 (0.2) |
| Multiple organ dysfunction syndrome | 0 | 1 (0.1) |
| Infections and infestations | 1 (0.1) | 1 (0.1) |
| Pneumonia | 1 (0.1) | 0 |
| Sepsis | 0 | 1 (0.1) |
| Injury, poisoning and procedural complications | 1 (0.1) | 0 |
| Craniocerebral injury | 1 (0.1) | 0 |
| Neoplasms benign, malignant and unspecified (including cysts and polyps) | 2 (0.2) | 0 |
| Cholangiocarcinoma | 1 (0.1) | 0 |
| Lung adenocarcinoma | 1 (0.1) | 0 |

Data presented as n (%).

The Safety set included two additional participants in the inclisiran arm (total n=900) compared to the Full Analysis Set, which had 898 participants.

**ioLLT, individually optimized lipid-lowering therapy.**

**Table S8. TESAE by primary system organ class and preferred term (safety set)**

| **Primary system organ class     Preferred term** | **Inclisiran-based treatment strategy, n=900** | **ioLLT, n=870** |
| --- | --- | --- |
| At least one TESAE | 148 (16.4) | 148 (17.0) |
| **Blood and lymphatic system disorders** | **2 (0.2)** | **2 (0.2)** |
| Immune thrombocytopenia | 1 (0.1) | 0 |
| Lymphadenopathy mediastinal | 1 (0.1) | 0 |
| Iron deficiency anaemia | 0 | 1 (0.1) |
| Polycythaemia | 0 | 1 (0.1) |
| **Cardiac disorders** | **52 (5.8)** | **33 (3.8)** |
| Atrial fibrillation | 12 (1.3) | 5 (0.6) |
| Angina unstable | 7 (0.8) | 2 (0.2) |
| Coronary artery disease | 7 (0.8) | 5 (0.6) |
| Angina pectoris | 5 (0.6) | 9 (1.0) |
| Acute coronary syndrome | 4 (0.4) | 1 (0.1) |
| Cardiac failure | 4 (0.4) | 2 (0.2) |
| Myocardial infarction | 4 (0.4) | 2 (0.2) |
| Ventricular tachycardia | 4 (0.4) | 1 (0.1) |
| Atrioventricular block | 3 (0.3) | 0 |
| Cardiac failure congestive | 3 (0.3) | 0 |
| Arrhythmia | 2 (0.2) | 0 |
| Acute myocardial infarction | 1 (0.1) | 3 (0.3) |
| Atrioventricular block second degree | 0 | 2 (0.2) |
| **Congenital, familial and genetic disorders** | **0** | **1 (0.1)** |
| **Ear and labyrinth disorders** | **2 (0.2)** | **0** |
| **Endocrine disorders** | **1 (0.1)** | **1 (0.1)** |
| **Eye disorders** | **1 (0.1)** | **2 (0.2)** |
| **Gastrointestinal disorders** | **4 (0.4)** | **12 (1.4)** |
| Gastrointestinal haemorrhage | 1 (0.1) | 2 (0.2) |
| Abdominal pain | 0 | 2 (0.2) |
| General disorders and administration site conditions | 10 (1.1) | 6 (0.7) |
| Chest pain | 5 (0.6) | 1 (0.1) |
| Pain | 2 (0.2) | 0 |
| Pyrexia | 2 (0.2) | 0 |
| Vascular stent stenosis | 2 (0.2) | 0 |
| Death | 0 | 2 (0.2) |
| **Hepatobiliary disorders** | **8 (0.9)** | **3 (0.3)** |
| Cholecystitis | 2 (0.2) | 2 (0.2) |
| Cholecystitis acute | 2 (0.2) | 0 |
| Cholelithiasis | 2 (0.2) | 1 (0.1) |
| **Immune system disorders** | **0** | **1 (0.1)** |
| **Infections and infestations** | **23 (2.6)** | **17 (2.0)** |
| Pneumonia | 6 (0.7) | 4 (0.5) |
| COVID-19 | 4 (0.4) | 3 (0.3) |
| COVID-19 pneumonia | 2 (0.2) | 0 |
| Urinary tract infection | 2 (0.2) | 0 |
| Appendicitis | 0 | 2 (0.2) |
| Erysipelas | 0 | 2 (0.2) |
| **Injury, poisoning and procedural complications** | **12 (1.3)** | **10 (1.1)** |
| Craniocerebral injury | 2 (0.2) | 0 |
| Tendon rupture | 2 (0.2) | 0 |
| Joint dislocation | 0 | 2 (0.2) |
| **Investigations** | **5 (0.6)** | **5 (0.6)** |
| Alanine aminotransferase increased | 0 | 2 (0.2) |
| Hepatic enzyme increased | 0 | 2 (0.2) |
| Metabolism and nutrition disorders | 2 (0.2) | 3 (0.3) |
| Hypoglycemia | 0 | 2 (0.2) |
| **Musculoskeletal and connective tissue disorders** | **16 (1.8)** | **20 (2.3)** |
| Osteoarthritis | 4 (0.4) | 12 (1.4) |
| Intervertebral disc protrusion | 2 (0.2) | 0 |
| Spinal stenosis | 2 (0.2) | 1 (0.1) |
| Arthritis | 0 | 2 (0.2) |
| Myalgia | 0 | 2 (0.2) |
| **Neoplasms benign, malignant and unspecified (incl cysts and polyps)** | **8 (0.9)** | **16 (1.8)** |
| Adenocarcinoma of colon | 0 | 2 (0.2) |
| Bladder cancer | 0 | 2 (0.2) |
| **Nervous system disorders** | **13 (1.4)** | **18 (2.1)** |
| Syncope | 3 (0.3) | 2 (0.2) |
| Cerebrovascular accident | 2 (0.2) | 2 (0.2) |
| Carotid artery stenosis | 1 (0.1) | 2 (0.2) |
| Ischemic stroke | 1 (0.1) | 4 (0.5) |
| Transient ischemic attack | 1 (0.1) | 2 (0.2) |
| Sciatica | 0 | 2 (0.2) |
| **Product issues** | **1 (0.1)** | **1 (0.1)** |
| **Psychiatric disorders** | **2 (0.2)** | **3 (0.3)** |
| **Renal and urinary disorders** | **5 (0.6)** | **9 (1.0)** |
| Acute kidney injury | 2 (0.2) | 4 (0.5) |
| **Reproductive system and breast disorders** | **0** | **2 (0.2)** |
| **Respiratory, thoracic and mediastinal disorders** | **12 (1.3)** | **4 (0.5)** |
| Dyspnoea | 3 (0.3) | 0 |
| Dyspnoea exertional | 2 (0.2) | 0 |
| Obstructive sleep apnoea syndrome | 2 (0.2) | 0 |
| Sleep apnoea syndrome | 2 (0.2) | 0 |
| Pulmonary embolism | 1 (0.1) | 2 (0.2) |
| **Skin and subcutaneous tissue disorders** | **1 (0.1)** | **3 (0.3)** |
| Vascular disorders | 13 (1.4) | 18 (2.1) |
| Peripheral arterial occlusive disease | 7 (0.8) | 6 (0.7) |
| Hypertensive crisis | 2 (0.2) | 1 (0.1) |
| Aortic aneurysm | 0 | 4 (0.5) |

Data presented as n (%). AEs occurring in 0.2% of participants in any treatment arm are reported.

The safety set included two additional participants in the inclisiran arm (total n=900) compared to the Full Analysis Set, which had 898 participants.

**ioLLT, individually optimized lipid-lowering therapy**.

# **SUPPLEMENTARY FIGURE LEGENDS**

**Figure S1. Participant disposition**

**The full analysis set included 1770 participants as 6 were mis-randomized (inclisiran-based treatment strategy, n=4; ioLLT, n=2).**

**ioLLT, individually optimized lipid-lowering therapy.**

**Figure S2. Percentage of participants achieving their LDL-C goals per visit in the VICTORION-Difference study (safety set)**

**ioLLT, individually optimised lipid-lowering therapy;** LDL-C, **low-density lipoprotein cholesterol;.**

**Figure S3.** **Annualized rate (95% CI) of days with pain from Day 1 to Day 360 in the VICTORION-Difference study population (full analysis set)**

*1-sided p-value (the difference was not statistically significant)

CI, confidence interval, ioLLT, individually optimised lipid-lowering therapy; M, number of patients at risk, i.e. patients completing the diary at least once.

Annualized rate refers to the estimated number of days with pain per year.

**Figure S4. Percentage of participants on MTD of rosuvastatin by visit and treatment in the VICTORION-Difference study (safety set)**

ioLLT, individually optimized lipid-lowering therapy; LDL-C, **low-density lipoprotein cholesterol; MTD, maximally tolerated dose.**

**Figure S5. Rosuvastatin dose level by visit and treatment in the VICTORION-Difference study**

EOS, end of study; ioLLT, individually optimized lipid-lowering therapy; LDL-C, **low-density lipoprotein cholesterol.**

**Figure S6. Summary of additional LLTs for escalation by visit and treatment in the VICTORION-Difference study**

ioLLT, individually optimized LLT; LDL-C, **low-density lipoprotein cholesterol; LLT, lipid lowering therapy; mAb, monoclonal antibody;** PCSK9, proprotein convertase subtilisin/kexin type 9**.**

# **SUPPLEMENTARY FIGURES**

**Figure S1. Participant disposition**


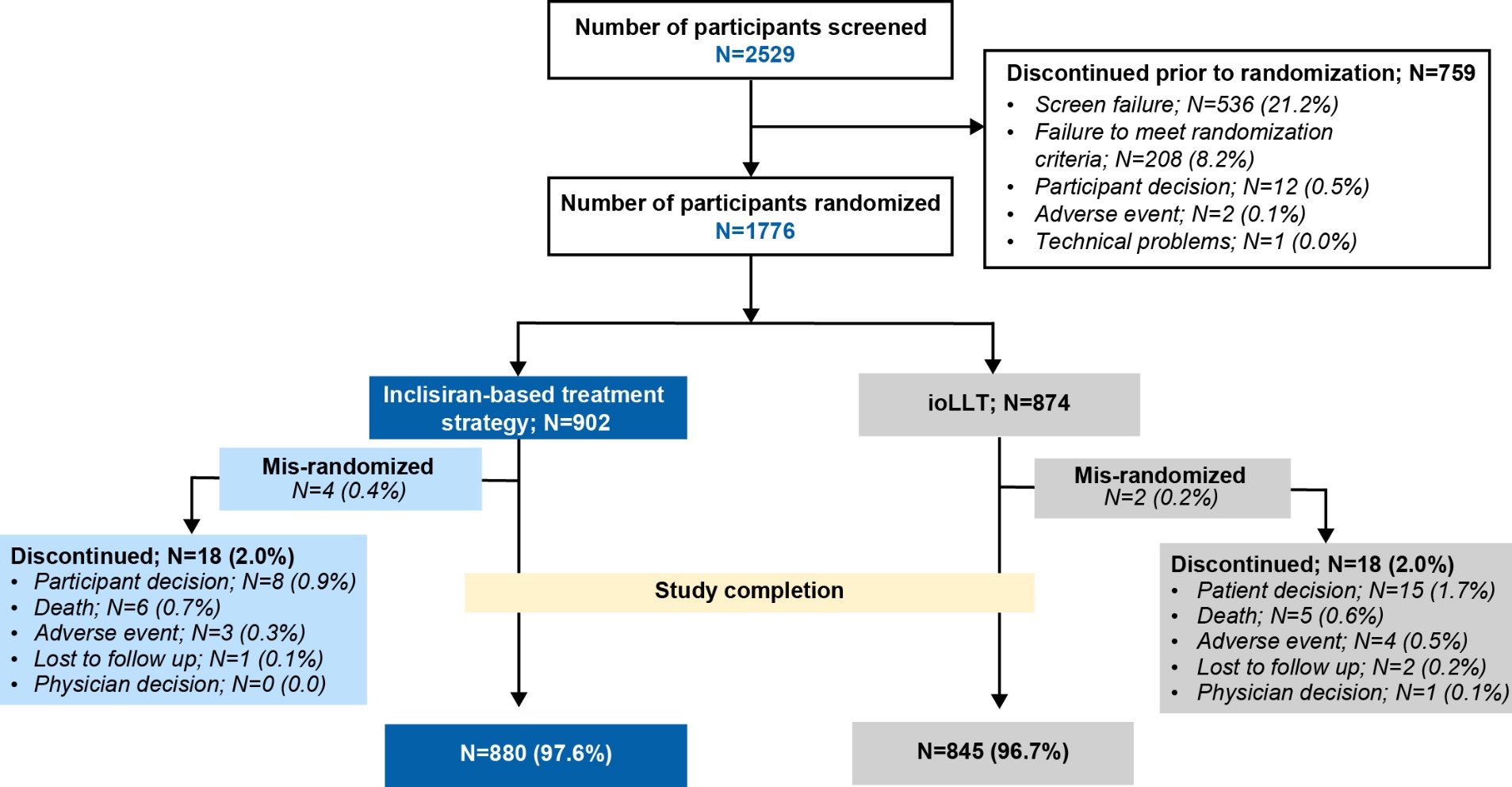


**The full analysis set included 1770 participants as 6 were mis-randomized (inclisiran-based treatment strategy, n=4; ioLLT, n=2).**

**ioLLT, individually optimized lipid-lowering therapy.**

**Figure S2. Percentage of participants achieving their LDL-C goals per visit in the VICTORION-Difference study (safety set)**


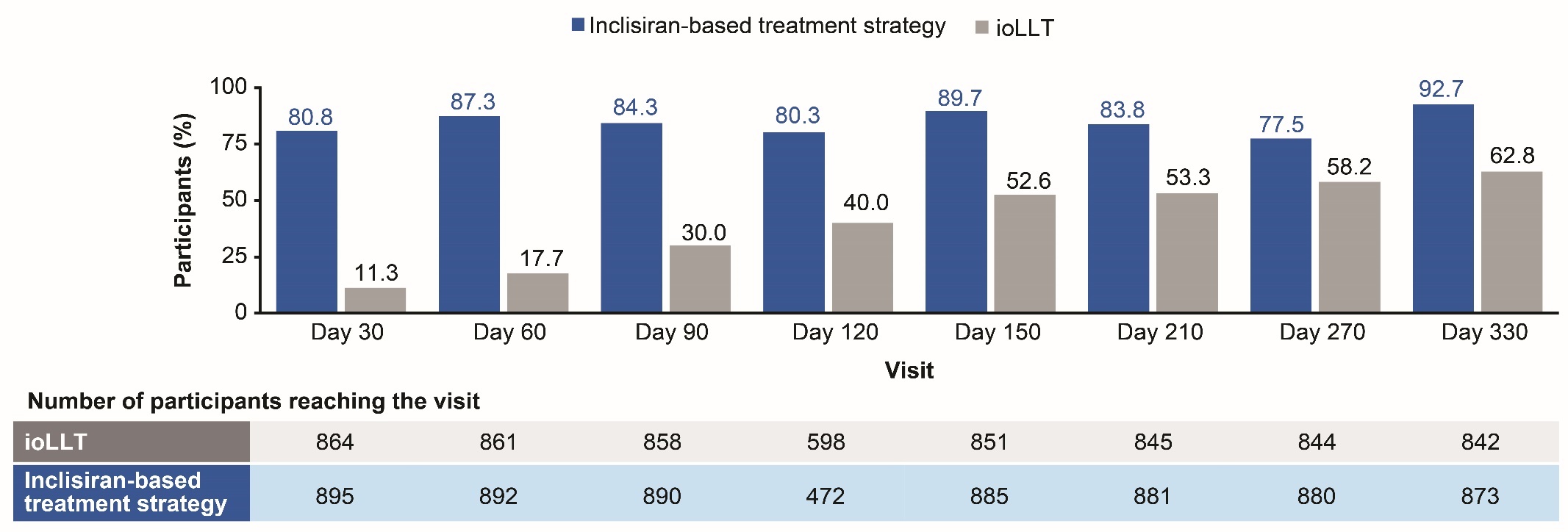


**ioLLT, individually optimized lipid-lowering therapy;** LDL-C, **low-density lipoprotein cholesterol.**

**Figure S3.** **Annualized rate (95% CI) of days with pain from Day 1 to Day 360 in the VICTORION-Difference study population (full analysis set)**


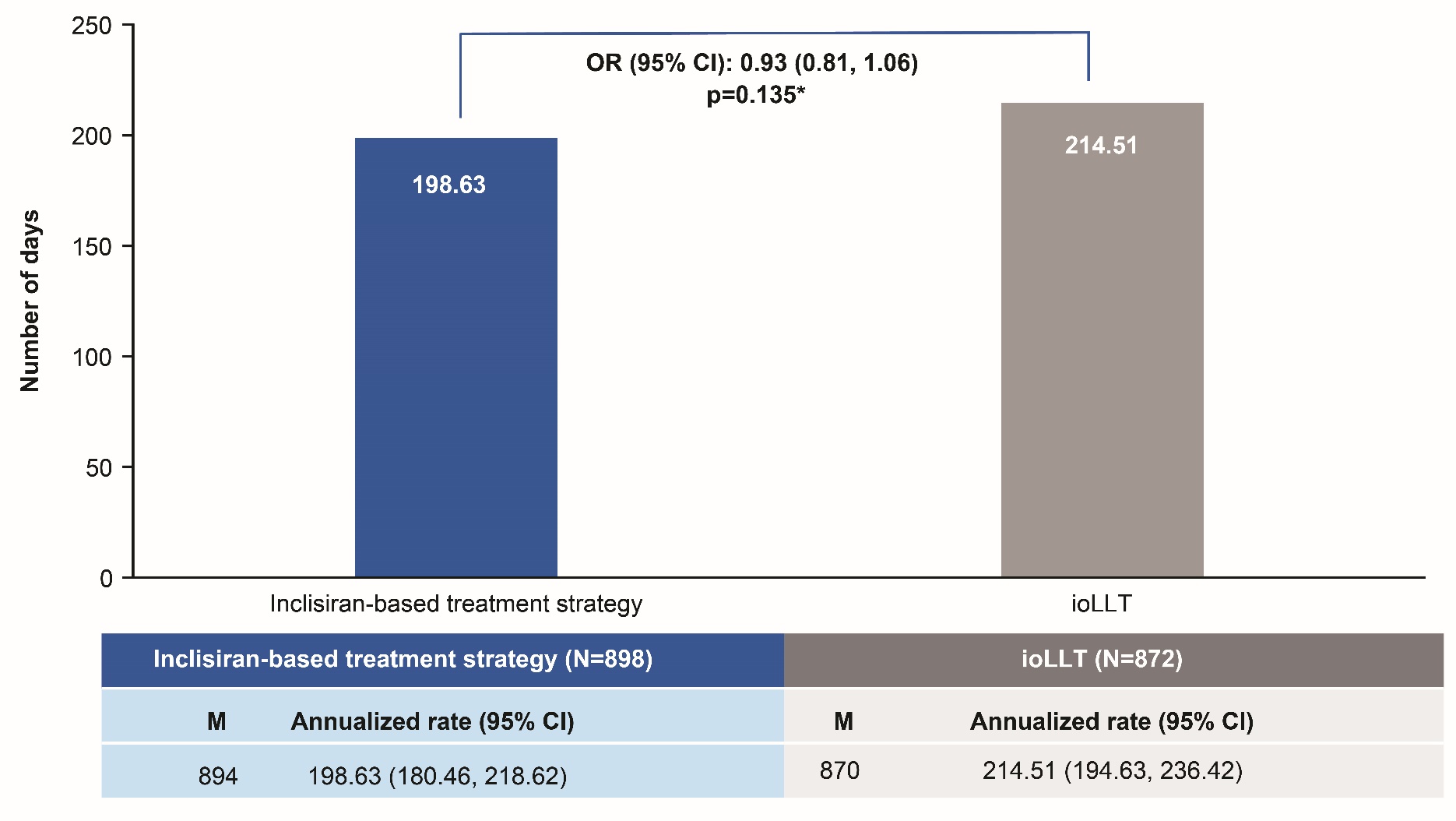


*1-sided p-value (the difference was not statistically significant)

CI, confidence interval, ioLLT, individually optimized lipid-lowering therapy; M, number of patients at risk, i.e. patients completing the diary at least once.

Annualized rate refers to the estimated number of days with pain per year.

**Figure S4. Percentage of participants on MTD of rosuvastatin by visit and treatment in the VICTORION-Difference study (safety set)**

**
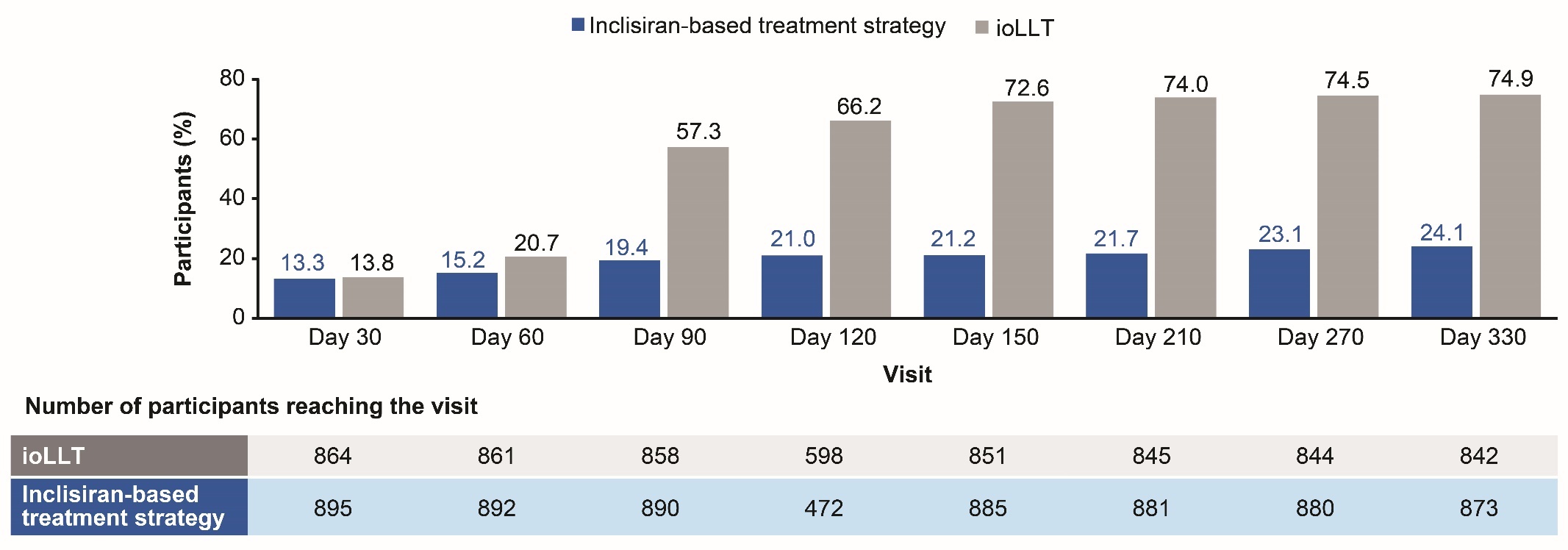
**

ioLLT, individually optimized lipid-lowering therapy; LDL-C, **low-density lipoprotein cholesterol; MTD, maximally tolerated dose**

**Figure S5. Rosuvastatin dose level by visit and treatment in the VICTORION-Difference study**


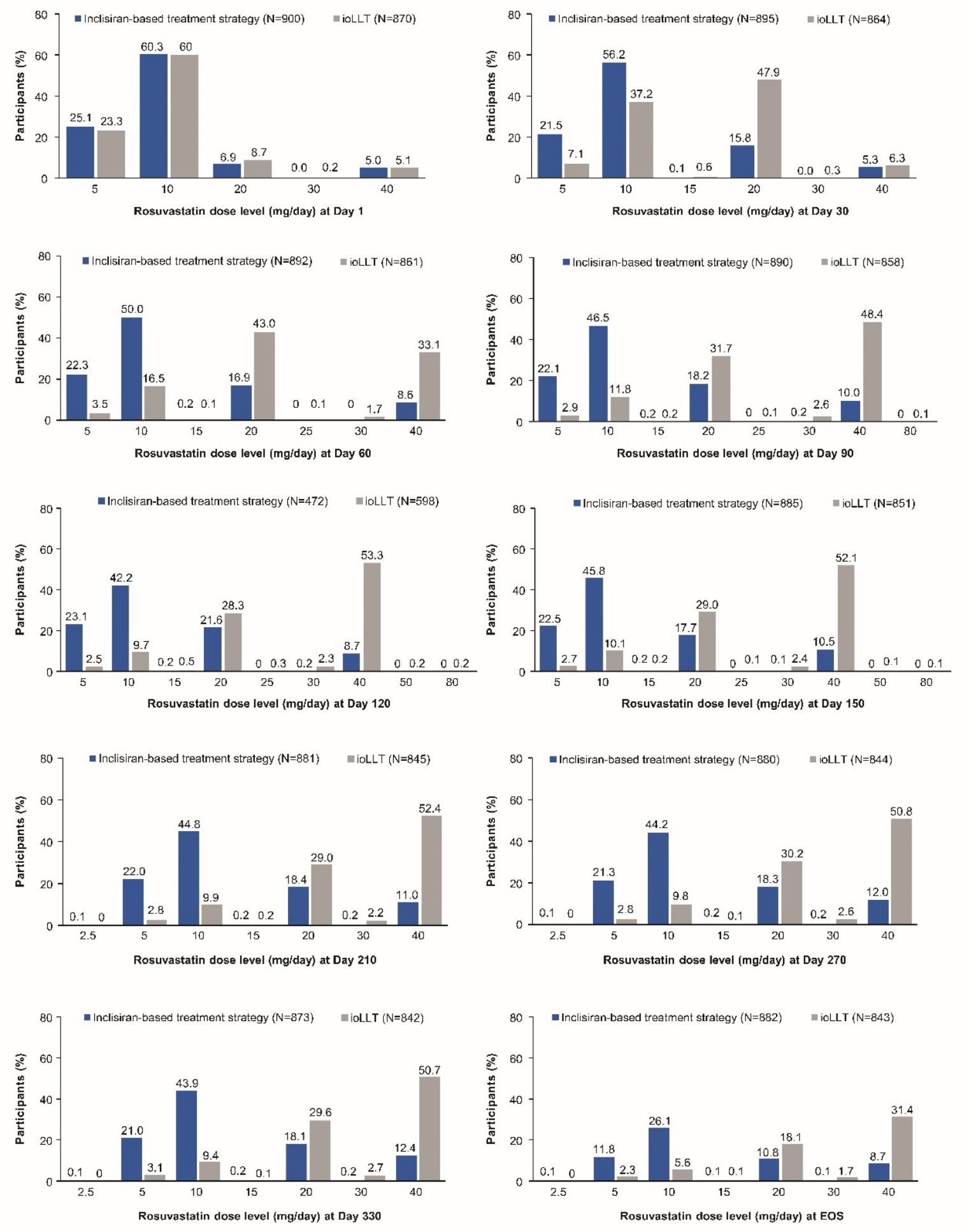


EOS, end of study; ioLLT, individually optimized lipid-lowering therapy; LDL-C, **low-density lipoprotein cholesterol.**

**Figure S6. Summary of additional LLTs for escalation by visit and treatment in the VICTORION-Difference study**

**
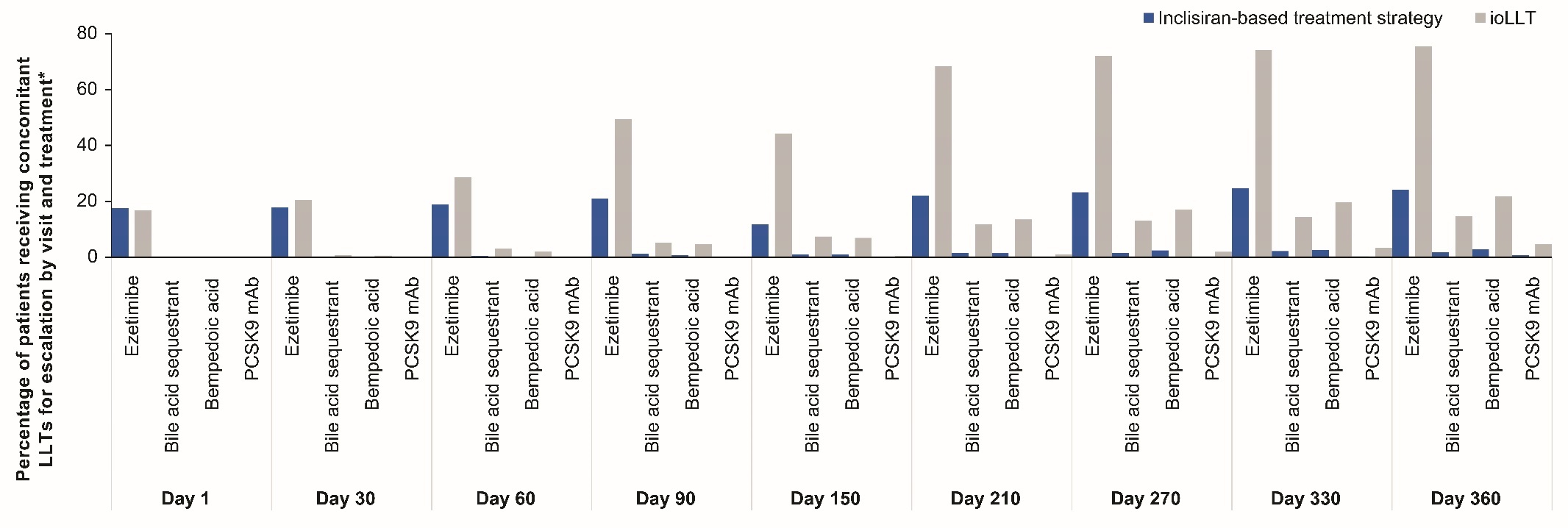
**

*Both treatment arms received simultaneous LLT optimization.

ioLLT, individually optimized LLT; LDL-C, **low-density lipoprotein cholesterol; LLT, lipid lowering therapy; mAb, monoclonal antibody;** PCSK9, proprotein convertase subtilisin/kexin type 9**.**

**REFERENCES**

1. Landmesser U, Laufs U, Schatz U, Winzer EB, Nowak B, Kassner U, et al. Design and rationale of the VICTORION-Difference study: A phase 4 randomized, double-blind, placebo-controlled clinical trial to assess inclisiran's early efficacy, safety, tolerability, as well as its impact on quality of life in individuals with hypercholesterolemia. *Am Heart J* 2025;**289**:117-126. doi: <https://doi.org/10.1016/j.ahj.2025.05.014>

2. Keller S, Bann CM, Dodd SL, Schein J, Mendoza TR, Cleeland CS. Validity of the brief pain inventory for use in documenting the outcomes of patients with noncancer pain. *Clin J Pain* 2004;**20**:309-318. doi: <https://doi.org/10.1097/00002508-200409000-00005>

3. Tan G, Nguyen Q, Anderson KO, Jensen M, Thornby J. Further validation of the chronic pain coping inventory. *J Pain* 2005;**6**:29-40. doi: <https://doi.org/10.1016/j.jpain.2004.09.006>

4. Cleeland CS. *The Brief Pain Inventory User Guide* <https://www.mdanderson.org/documents/Departments-and-Divisions/Symptom-Research/BPI_UserGuide.pdf> (June 20 2025)

5. Ware JE, Jr., Sherbourne CD. The MOS 36-item short-form health survey (SF-36). I. Conceptual framework and item selection. *Med Care* 1992;**30**:473-483.

6. Ware JE, Jr. Hypertension--when is the clinical problem solved? When quality of life is secured. *Cardiology* 1994;**85 Suppl 1**:65-70. doi: <https://doi.org/10.1159/000176762>

7. Hyttinen L, Kekalainen P, Vuorio AF, Sintonen H, Strandberg TE. Health-related quality of life in elderly patients with familial hypercholesterolemia. *Int J Technol Assess Health Care* 2008;**24**:228-234. doi: <https://doi.org/10.1017/S0266462308080318>

8. Muller-Nordhorn J, Roll S, Willich SN. Comparison of the short form (SF)-12 health status instrument with the SF-36 in patients with coronary heart disease. *Heart* 2004;**90**:523-527. doi: <https://doi.org/10.1136/hrt.2003.013995>

9. Poku E, Duncan R, Keetharuth A, Essat M, Phillips P, Woods HB, et al. Patient-reported outcome measures in patients with peripheral arterial disease: a systematic review of psychometric properties. *Health Qual Life Outcomes* 2016;**14**:161. doi: <https://doi.org/10.1186/s12955-016-0563-y>

10. Dempster M, Donnelly M. Measuring the health related quality of life of people with ischaemic heart disease. *Heart* 2000;**83**:641-644. doi: <https://doi.org/10.1136/heart.83.6.641>

11. Ware JE, Jr., Gandek B. Overview of the SF-36 Health Survey and the International Quality of Life Assessment (IQOLA) Project. *J Clin Epidemiol* 1998;**51**:903-912. doi: <https://doi.org/10.1016/s0895-4356(98)00081-x>

12. Alonso J, Ferrer M, Gandek B, Ware JE, Jr., Aaronson NK, Mosconi P, et al. Health-related quality of life associated with chronic conditions in eight countries: results from the International Quality of Life Assessment (IQOLA) Project. *Qual Life Res* 2004;**13**:283-298. doi: <https://doi.org/10.1023/b:qure.0000018472.46236.05>

13. Huber A, Oldridge N, Hofer S. International SF-36 reference values in patients with ischemic heart disease. *Qual Life Res* 2016;**25**:2787-2798. doi: <https://doi.org/10.1007/s11136-016-1316-4>

14. Svedlund J, Sjodin I, Dotevall G. GSRS--a clinical rating scale for gastrointestinal symptoms in patients with irritable bowel syndrome and peptic ulcer disease. *Dig Dis Sci* 1988;**33**:129-134. doi: <https://doi.org/10.1007/BF01535722>

15. Chan L, Mulgaonkar S, Walker R, Arns W, Ambuhl P, Schiavelli R. Patient-reported gastrointestinal symptom burden and health-related quality of life following conversion from mycophenolate mofetil to enteric-coated mycophenolate sodium. *Transplantation* 2006;**81**:1290-1297. doi: <https://doi.org/10.1097/01.tp.0000209411.66790.b3>

16. Kulich KR, Malfertheiner P, Madisch A, Labenz J, Bayerdorffer E, Miehlke S, et al. Psychometric validation of the German translation of the Gastrointestinal Symptom Rating Scale (GSRS) and Quality of Life in Reflux and Dyspepsia (QOLRAD) questionnaire in patients with reflux disease. *Health Qual Life Outcomes* 2003;**1**:62. doi: <https://doi.org/10.1186/1477-7525-1-62>

17. Kulich KR, Madisch A, Pacini F, Pique JM, Regula J, Van Rensburg CJ, et al. Reliability and validity of the Gastrointestinal Symptom Rating Scale (GSRS) and Quality of Life in Reflux and Dyspepsia (QOLRAD) questionnaire in dyspepsia: a six-country study. *Health Qual Life Outcomes* 2008;**6**:12. doi: <https://doi.org/10.1186/1477-7525-6-12>

18. Revicki DA, Wood M, Wiklund I, Crawley J. Reliability and validity of the Gastrointestinal Symptom Rating Scale in patients with gastroesophageal reflux disease. *Qual Life Res* 1998;**7**:75-83. doi: <https://doi.org/10.1023/a:1008841022998>

19. Samaha FF, McKenney J, Bloedon LT, Sasiela WJ, Rader DJ. Inhibition of microsomal triglyceride transfer protein alone or with ezetimibe in patients with moderate hypercholesterolemia. *Nat Clin Pract Cardiovasc Med* 2008;**5**:497-505. doi: <https://doi.org/10.1038/ncpcardio1250>

20. Dimenas E, Glise H, Hallerback B, Hernqvist H, Svedlund J, Wiklund I. Well-being and gastrointestinal symptoms among patients referred to endoscopy owing to suspected duodenal ulcer. *Scand J Gastroenterol* 1995;**30**:1046-1052. doi: <https://doi.org/10.3109/00365529509101605>

21. Atkinson MJ, Kumar R, Cappelleri JC, Hass SL. Hierarchical construct validity of the treatment satisfaction questionnaire for medication (TSQM version II) among outpatient pharmacy consumers. *Value Health* 2005;**8 Suppl 1**:S9-S24. doi: <https://doi.org/10.1111/j.1524-4733.2005.00066.x>

22. Atkinson MJ, Sinha A, Hass SL, Colman SS, Kumar RN, Brod M, et al. Validation of a general measure of treatment satisfaction, the Treatment Satisfaction Questionnaire for Medication (TSQM), using a national panel study of chronic disease. *Health Qual Life Outcomes* 2004;**2**:12. doi: <https://doi.org/10.1186/1477-7525-2-12>

23. Mulchandani R, Lyngdoh T, Chakraborty P, Kakkar AK. Satisfaction With Statin Treatment Among Adult Coronary Artery Disease Patients: An Experience From a Resource-Constrained Setting. *Heart Lung Circ* 2019;**28**:1788-1794. doi: <https://doi.org/10.1016/j.hlc.2018.10.024>

24. Rodriguez Arroyo LA, Diaz Rodriguez A, Pinto Sala X, Coca Payeras A, Rius Tarruella J. [Effectivity and satisfaction with the treatment for dyslipidemia with pitavastatin. Multicentric, descriptive, post authorised and observational study (REINA study)]. *Clin Investig Arterioscler* 2014;**26**:205-217. doi: <https://doi.org/10.1016/j.arteri.2014.01.005>

25. Regensteiner JG, Steiner JF, Hiatt WR. Exercise training improves functional status in patients with peripheral arterial disease. *J Vasc Surg* 1996;**23**:104-115. doi: <https://doi.org/10.1016/s0741-5214(05)80040-0>

26. Regensteiner JG, Steiner JF, Panzer RJ, Hiatt WR. Evaluation of walking impairment by questionnaire in patients with peripheral arterial disease. *J Vasc Med Biol* 1990;**2**:142‐152.

27. Nicolai SP, Kruidenier LM, Rouwet EV, Graffius K, Prins MH, Teijink JA. The walking impairment questionnaire: an effective tool to assess the effect of treatment in patients with intermittent claudication. *J Vasc Surg* 2009;**50**:89-94. doi: <https://doi.org/10.1016/j.jvs.2008.12.073>

28. McDermott MM, Ades P, Guralnik JM, Dyer A, Ferrucci L, Liu K, et al. Treadmill exercise and resistance training in patients with peripheral arterial disease with and without intermittent claudication: a randomized controlled trial. *JAMA* 2009;**301**:165-174. doi: <https://doi.org/10.1001/jama.2008.962>

29. Coyne KS, Margolis MK, Gilchrist KA, Grandy SP, Hiatt WR, Ratchford A, et al. Evaluating effects of method of administration on Walking Impairment Questionnaire. *J Vasc Surg* 2003;**38**:296-304. doi: <https://doi.org/10.1016/s0741-5214(03)00312-4>

30. McDermott MM, Liu K, Guralnik JM, Martin GJ, Criqui MH, Greenland P. Measurement of walking endurance and walking velocity with questionnaire: validation of the walking impairment questionnaire in men and women with peripheral arterial disease. *J Vasc Surg* 1998;**28**:1072-1081. doi: <https://doi.org/10.1016/s0741-5214(98)70034-5>

31. Sagar SP, Brown PM, Zelt DT, Pickett WL, Tranmer JE. Further clinical validation of the walking impairment questionnaire for classification of walking performance in patients with peripheral artery disease. *Int J Vasc Med* 2012;**2012**:190641. doi: <https://doi.org/10.1155/2012/190641>

32. Nead KT, Zhou M, Diaz Caceres R, Olin JW, Cooke JP, Leeper NJ. Walking impairment questionnaire improves mortality risk prediction models in a high-risk cohort independent of peripheral arterial disease status. *Circ Cardiovasc Qual Outcomes* 2013;**6**:255-261. doi: <https://doi.org/10.1161/CIRCOUTCOMES.111.000070>

33. Lim LL, Valenti LA, Knapp JC, Dobson AJ, Plotnikoff R, Higginbotham N, et al. A self-administered quality-of-life questionnaire after acute myocardial infarction. *J Clin Epidemiol* 1993;**46**:1249-1256. doi: <https://doi.org/10.1016/0895-4356(93)90089-j>

34. Valenti L, Lim L, Heller RF, Knapp J. An improved questionnaire for assessing quality of life after acute myocardial infarction. *Qual Life Res* 1996;**5**:151-161. doi: <https://doi.org/10.1007/BF00435980>

35. Hofer S, Lim L, Guyatt G, Oldridge N. The MacNew Heart Disease health-related quality of life instrument: a summary. *Health Qual Life Outcomes* 2004;**2**:3. doi: <https://doi.org/10.1186/1477-7525-2-3>

36. Hofer S, Saleem A, Stone J, Thomas R, Tulloch H, Oldridge N. The MacNew Heart Disease Health-Related Quality of Life Questionnaire in patients with angina and patients with ischemic heart failure. *Value Health* 2012;**15**:143-150. doi: <https://doi.org/10.1016/j.jval.2011.07.003>
